# Integrated energy management for enhanced grid flexibility: Optimizing renewable resources and energy storage systems across transmission and distribution networks

**DOI:** 10.1016/j.heliyon.2024.e39585

**Published:** 2024-10-18

**Authors:** Meysam Khani, Mahmoud Samiei Moghaddam, Tohid Noori, Reza Ebrahimi

**Affiliations:** a Department of Electrical Engineering, Sari Branch, Islamic Azad University, Sari, Iran; b Department of Electrical Engineering, Damghan Branch, Islamic Azad University, Damghan, Iran; c Department of Electrical Engineering, Gorgan Branch, Islamic Azad University, Gorgan, Iran

**Keywords:** Smart distribution grid, Transmission system, Bi-level optimization, Renewable resources, Energy storage system, Demand side management

## Abstract

This study explores the enhancement of electric grid flexibility and the realization of smart grid objectives through the integration of renewable energy (RE) resources and energy storage systems (ESS). While prior research has mainly concentrated on optimizing ESS operations either within the transmission network as a versatile power source or within the distribution network using small-scale batteries, our approach offers a more holistic perspective. We introduce a bi-level stochastic model for integrated energy management that encompasses renewable energy, demand side management (DSM), transmission, and distribution networks as interconnected entities. To tackle the binary variables inherent in the model, we employ a precise method based on reformulation and decomposition techniques to ensure globally optimal solutions. We evaluate the efficacy of our proposed model and the influence of ESS on the networks using various integrated transmission and distribution network systems. Our findings demonstrate the model's efficiency and underscore the cost-saving benefits of integrating energy storage systems. Specifically, incorporating ESS into the distribution grid results in a 13 % reduction in distribution network costs, while deploying large batteries in the transmission grid leads to an impressive 83 % cost reduction.

## Highlights

• Integrated energy management enhances flexibility of transmission and distribution grids.
• Bi-level stochastic model optimizes renewable energy and storage systems integration.
• Reformulation and decomposition techniques ensure globally optimal solutions.
• ESS in distribution grids cuts costs by 13 %, in transmission grids by 83 %.
• Demand side management integrates with ESS for holistic grid optimization.

**Table:** Nomenclature

Indexes and sets:	
*N, n*	Set and index of transmission and distribution bus, respectively.
*T, t*	Set and index of time, respectively.
*S, s*	Set and index of scenario, respectively.
*L, nm*	Set and index of transmission and distribution line, respectively.
Variables:	
$G_{n,t}$	The power produced by the unit in the *n*th bus at time *t*.
$u_{n,t},s_{n,t},v_{n,t}$	Binary variable related to the status of the units, startup and shutdown, respectively.
$r_{n,t}$	The power curtailment in the *n*th bus at time *t*.
$f_{nm,t}$	The real power flow from bus *n* to *m*.
$P_{n,t}^{s}$، $Q_{n,t}^{s}$	The active and reactive power of the *n*th distribution network substation at time *t*, respectively.
$z_{n,t}$	Binary variable related to security constraints.
$P_{n,t}^{DG}$	The real power produced by distributed generation in bus *n* of the distribution network at time *t*.
$f_{nm,t}^{p}$، $f_{nm,t}^{q}$	The real and reactive power flux of the nm line of the distribution network, respectively.
$V_{n,t}^{sqe}$	The square voltage of the bus *n* of the distribution network at time *t*.
${\tilde{d}}_{n,t}^{p}$، ${\tilde{d}}_{n,t}^{q}$	Changed real and reactive power in the demand side management program at bus *n* and time *t*, respectively.
$p_{n,t}^{dis},p_{n,t}^{ch}$	Discharging and charging power of the battery in bus *n* at time *t*, respectively.
$e_{n,t}^{ess}$	Battery energy in the bus *n* at time *t*.
$\zeta_{n,t}^{ess}$	Charging and discharging status of the battery in bus *n* at time *t*.
$P_{n,t}^{PV}$	The real power produced by PV generation in bus *n* of the distribution network at time *t*.
$P_{n,t}^{PEV}$	The real power produced by EV in bus *n* of the distribution network at time *t*.
Parameters:	
$c_{n}^{g}$	The unit production cost in bus *n.*
$c_{n}^{c}$	The cost of no-load unit in bus *n.*
$c_{n}^{d}$	The shutdown cost of the unit in bus *n*.
$c_{n}^{r}$	The cost of load curtailment at the bus *n*.
$c_{t}^{loss}$	The cost of power losses.
$D_{n,t}$	Active load on bus *n* at time *t*.
$\underline{G_{n}}$، $\bar{G_{n}}$	The minimum and maximum power of the units, respectively.
$\bar{f_{nm}}$	Maximum power flow from bus *n* to *m*.
$\bar{R_{n}}$، $\underline{R_{n}}$	Maximum and minimum ramp units, respectively.
$c_{t}^{s}$	The cost of purchasing energy from the transmission network.
$c_{n}^{RN}$، $c_{n}^{PEV}$	The cost of non-participation of renewable resources and electric vehicle stations, respectively.
${\bar{P}}_{n,t}^{RN}$، ${\bar{P}}_{n,t}^{PEV}$	The actual value of the active power of renewable resources and the actual charging power required by electric vehicle charging stations, respectively.
$c_{n}^{DG}$	Fuel cost of gas-fired distributed generations.
$d_{n,t}^{p}$، $d_{n,t}^{q}$	Real and reactive load in the *n*th bus of the distribution network at time *t*, respectively.
$\rho_{n}$	Power factor of the non-renewable distributed generation resources.
${\bar{f}}_{nm}^{p}$، ${\underline{f}}_{nm}^{p}$	Maximum and minimum active power flow through the *nm* line of the distribution network, respectively.
${\bar{f}}_{nm}^{q}$، ${\underline{f}}_{nm}^{q}$	Maximum and minimum reactive power flow through the *nm* line of the distribution network, respectively.
$r_{nm}$، $x_{nm}$	The resistance and reactance of the *nm* line of the distribution network, respectively.
$V_{n}^{\min}$، $V_{n}^{\max}$	The minimum and maximum voltage of the *n*th bus of the distribution network, respectively.
$X_{n}^{ess}$	Battery capacity in the *n*th bus.
$\eta_{n}^{ess}$	Battery efficiency at bus *n*.
A	The maximum number of times allowed discharging the battery in one day.
ε	The percentage of load changes in the demand side management program.
$\sigma_{s}$	Probability of the scenario *s*.
Abbreviations:	
TSO	Transmission system operators.
DSO	Distribution system operators.
RDG	Renewable distributed generation.
ESS	Energy storage systems.
SCUC	Security-constrained unit commitment.
MO	Multi-objective.
PHEV	Plug-in hybrid electric vehicles.
EV	Electric vehicles.
DG	Distributed generation.
CHP	Combined heat and power.
DNB	Distribution network battery.
TNB	Transmission network battery.
UC	Unit Commitment.
RDG	Renewable DG.
NLP	Non-linear programming.
LP	Linear programming.
RO	Robust optimization.
SOCP	Second-order cone programming.
MILP	Mixed-integer linear program.
MISOCP	Mixed-integer second-order cone programming.
CCP	Chance-constrained programming.
MIQCP	Mixed integer quadratically constrained program.
MINLP	Mixed integer non-linear programming.
ACOPF	AC optimal power flow.
OCO	Online convex optimization.
KKT	Karush–Kuhn–Tucker.
DSM	Demand-side management.
PV	Photovoltaic.

## 1 Introduction

Modern power systems vary in how countries define the roles of transmission system operators (TSOs) and distribution system operators (DSOs). As renewable distributed generation (RDG) and smart devices become more prevalent, efficient coordination between transmission and distribution networks is crucial. Energy storage systems (ESS) are increasingly important due to their flexibility and cost-effectiveness, serving vital functions in both networks. While many studies have optimized ESS operations at either level, few have explored their combined effects. This study introduces a novel bi-level model for simultaneous ESS optimization across both networks, accounting for security-constrained unit commitment (SCUC) in transmission and demand-side management in distribution. The proposed decomposition algorithm addresses the complexity of integer and binary variables, enhancing coordination between TSOs and DSOs. This approach improves system efficiency, reduces costs, and bolsters grid reliability and resilience, playing a critical role in maximizing the potential of renewable energy sources and achieving smart grid objectives.

In study [1], the authors propose an affine arithmetic-based method for coordinated interval power flow, improving the accuracy of power flow calculations in integrated transmission and distribution networks. In Ref. [2], the authors introduce the Generalized Master–Slave-Splitting method to address coordinated energy management [3] between transmission and distribution systems [4], ensuring efficient resource allocation. Reference [5] presents a robust reserve scheduling model, focusing on fully distributed solutions to optimize the operation of coupled transmission and distribution systems under uncertainty. In Ref. [6], the study examines the feasibility of California achieving a 50 % renewable grid [7], analyzing key challenges and strategies for integrating renewable resources. In Ref. [8], a decentralized AC optimal power flow (OPF) method is developed for integrated transmission and distribution grids, enabling better coordination between the two systems. Reference [9] presents a bi-level optimization model for pricing flexibility in active distribution networks, incorporating renewable energy and flexibility sources. In Ref. [10], a distributed restoration framework is proposed for integrated transmission and distribution systems with distributed energy resources (DERs), enhancing grid reliability during outages. In Ref. [11], the study integrates energy management for autonomous smart grids [12] into electricity market operations, proposing a coordinated scheme to optimize resource allocation. Reference [13] develops a stochastic market operation model for the coordinated management of transmission and distribution systems, addressing uncertainties in energy generation and consumption. In Ref. [14], a distributed control approach is used to manage battery [15] energy storage systems for voltage regulation at the transmission–distribution network interconnection points. In Ref. [16], a bi-level optimization model is proposed to coordinate the interaction between transmission and distribution systems and local energy markets, optimizing resource dispatch. In Ref. [17], a decentralized scheme is presented for the coordinated restoration of transmission and distribution systems, improving system recovery after disruptions. In Ref. [18], the study introduces a heterogeneous decomposition method based on LMP-sensitivity to coordinate economic dispatch between transmission and distribution networks. Reference [19] addresses the optimal scheduling of coupled transmission–distribution integrated electricity–gas systems using a distributed optimization approach [20] to improve energy management. In Ref. [21], a generic resource allocation method is introduced for the distribution grid, optimizing the deployment of resources across the network. In Ref. [22], an energy storage siting and sizing model is developed for coordinated transmission and distribution systems, ensuring optimal placement and capacity of energy storage systems [23]. In Ref. [24], the authors propose a novel once-data-exchange method to solve the coordinated ACOPF problem between transmission and distribution networks efficiently. In Ref. [25], the economic profit enhancement of demand response aggregators is explored through large-scale energy storage system investments, focusing on distribution grid profitability. Reference [26] presents a decentralized framework for coordinated multiperiod economic dispatch of transmission and distribution systems, optimizing energy flow over multiple periods. In Ref. [27], an asynchronous distributed global power flow method is developed, accounting for communication conditions in the coordinated analysis of transmission and distribution networks. In Ref. [28], the FERC and NERC report investigates the causes and recommendations of the Arizona-California outage in 2011, offering insights into improving grid resilience. In Ref. [29], the study proposes a learning-accelerated asynchronous decentralized optimization framework for integrated transmission and distribution systems, improving operational efficiency. In Ref. [30], a distributed stochastic unit commitment model is introduced to ensure the secure and coordinated operation of transmission and distribution systems. Reference [31] presents a local energy market clearing method that coordinates an active distribution network with a district heating network and the transmission system. In Ref. [32], a bilevel model is developed for security-constrained energy management of transmission and distribution substations, considering large-scale energy storage and demand-side management. In Ref. [33], a day-ahead optimal scheduling model is presented for integrated electricity–gas systems, using convex optimization to manage energy flow in transmission and distribution networks. In Ref. [34], the co-optimization of battery storage investment and grid expansion is analyzed for integrated energy systems, balancing economic and operational constraints. Reference [35] proposes a chance-constrained optimization framework for managing transmission congestion and frequency regulation, considering wind farms and energy storage systems. In Ref. [36], a hybrid Particle Swarm Optimization-Model Predictive Control (CPSO-MPC) algorithm is applied to optimize energy management for storage in microgrids. In Ref. [37], the authors propose a co-optimization model for velocity planning and energy management in autonomous plug-in hybrid electric vehicles, focusing on urban driving scenarios. Reference [38] introduces an improved monarch butterfly optimization method to manage transmission congestion through the rescheduling of generators. In Ref. [39], an initial-point strategy is developed for optimizing distribution system reconfiguration, improving grid efficiency and resilience. In Ref. [40], a bilevel optimization approach using evolutionary algorithms is introduced, focusing on approximating lower-level optimal solutions. Reference [41] discusses solving bilevel mixed integer programs through reformulation and decomposition techniques, enhancing optimization accuracy. In Ref. [42], a column-and-constraint generation method is employed to solve two-stage robust optimization problems, focusing on decision-making under uncertainty. Reference [43] provides the Gurobi Optimizer manual, offering guidance on solving various mathematical optimization problems using Gurobi software. In Ref. [44], a bi-level stochastic optimization model is developed for coordinating transmission and distribution networks, considering generic resource allocation. Reference [45] presents a market clearing price-based energy management framework for grid-connected renewable energy hubs [46] with flexible energy sources such as hydrogen and compressed air storage. In Ref. [47], the study explores uncertainty modeling for economic energy management in flexi-renewable energy hubs using the unscented transformation method. In Ref. [48], the economic design of renewable off-grid systems is optimized using compressed air energy storage, addressing both electricity and heat demand alongside smart charging [49] electric vehicles. Reference [50] explores the participation of energy storage-based [51] hubs in day-ahead reserve and energy markets, focusing on coordinated energy management strategies. In Ref. [52], a network-constrained unit commitment model is developed for virtual power plants in the day-ahead market, focusing on energy management optimization. Reference [53] proposes a bi-level fuzzy stochastic-robust model for valorizing flexibility in renewable networked microgrids [54], optimizing system reliability and cost efficiency. In Ref. [55], a flexibility pricing model is introduced for electric spring units and EV parking in microgrids, addressing both demand response and energy management challenges.

Despite advancements in ESS, there is still a gap in addressing the combined impact of ESS on both transmission and distribution networks. Most studies treat these levels separately, overlooking the benefits of a coordinated approach. This paper introduces a novel bi-level stochastic model that optimizes ESS operation for both networks. It integrates SCUC at the transmission level and demand-side management (DSM) at the distribution level, using decomposition algorithms to solve the complex problem. The study aims to enhance grid flexibility, reduce costs, and improve reliability through coordinated ESS operation. The objectives of the research are to develop an exact decomposition algorithm to solve the bi-level stochastic model, validate the benefits of coordinated ESS operation across both networks, and demonstrate improvements in grid flexibility, cost reduction, and reliability through detailed test cases. Table 1 highlights how this study differs from previous research by providing a more comprehensive modeling approach. Unlike earlier work that focused on areas like cost minimization and renewable energy integration, this paper models ESS at both the transmission and distribution levels, offering a holistic and efficient solution for modern power grids.

**Table 1:** Comparison between the proposed model and similar papers.

Study	Model	TNB	DNB	EV	DG	RDG	MO	Stochastic	UC	SCUC
[1]	NLP	˟	˟	˟	✓	✓	✓	˟	˟	˟
[2]	Bi-LP	˟	˟	˟	✓	✓	✓	˟	˟	✓
[5]	Bi-RO	˟	˟	˟	✓	✓	✓	˟	✓	˟
[6]	NLP	˟	˟	✓	✓	✓	˟	˟	˟	˟
[8]	Bi-SOCP	˟	˟	˟	✓	˟	˟	˟	˟	˟
[9]	Bi-MILP	˟	✓	˟	✓	✓	✓	✓	˟	˟
[10]	ACOPF	˟	˟	˟	✓	✓	✓	˟	˟	˟
[11]	Bi-MISOCP	˟	✓	˟	✓	✓	˟	˟	✓	˟
[13]	Bi-SOCP	˟	˟	˟	✓	✓	˟	✓	✓	˟
[14]	OCO	˟	✓	✓	✓	˟	✓	˟	˟	˟
[16]	Bi-MILP	˟	˟	˟	✓	✓	✓	˟	˟	˟
[17]	Bi-MIQP	˟	˟	˟	✓	✓	✓	˟	˟	✓
[18]	LP	˟	˟	˟	✓	˟	✓	˟	˟	˟
[19]	Bi-CCP	˟	˟	✓	✓	✓	✓	✓	˟	˟
[21]	MILP	˟	˟	˟	✓	✓	✓	˟	˟	˟
[22]	Bi-MIQP	˟	✓	˟	✓	✓	✓	˟	˟	˟
[24]	Bi-SOCP	˟	˟	˟	✓	˟	˟	˟	˟	˟
[25]	MILP	˟	˟	˟	✓	✓	✓	˟	˟	˟
[26]	Bi-MISOCP	˟	˟	✓	✓	✓	✓	˟	˟	✓
[27]	Bi-DNE	˟	˟	˟	✓	˟	✓	˟	˟	˟
[28]	Bi-MILP	˟	˟	✓	✓	✓	✓	˟	˟	˟
[29]	Bi-MIQP	˟	˟	˟	✓	✓	✓	✓	˟	˟
[30]	Bi-MIQP	˟	˟	˟	✓	✓	˟	✓	✓	✓
[31]	Bi-MISOCP	˟	˟	˟	✓	✓	✓	˟	˟	˟
[32]	Bi-MILP	˟	✓	˟	˟	˟	✓	˟	✓	✓
[33]	SOCP	˟	✓	˟	✓	✓	✓	˟	˟	˟
[34]	Bi-MILP	✓	˟	˟	˟	✓	✓	✓	✓	˟
[35]	MIQCP	✓	˟	˟	✓	✓	✓	˟	✓	˟
[36]	LP	✓	˟	˟	˟	✓	✓	˟	˟	˟
[37]	NLP	✓	˟	✓	˟	✓	˟	˟	˟	˟
[38]	MINLP	˟	˟	˟	✓	✓	✓	˟	˟	˟
[39]	NLP	˟	˟	˟	✓	✓	˟	˟	˟	˟
[44]	KKT	✓	✓	✓	✓	✓	✓	✓	✓	˟
Proposed	Bi-MILP	✓	✓	✓	✓	✓	✓	✓	✓	✓

This research tackles the challenges of solving mixed-integer bi-level models with binary variables at both upper and lower levels. Existing methods struggle with these complexities, so we propose an algorithm based on decomposition and reformulation techniques to address these issues. A key feature is the simultaneous modeling of ESS in both transmission and distribution networks, which previous studies have treated separately. Our approach integrates ESS coordination across both networks, incorporating electric vehicles, distributed generation, renewable energy, and SCUC. This unified model fills a crucial gap by optimizing coordination between transmission and distribution operators. Our algorithm ensures global optimal solutions while enhancing computational efficiency and scalability for real-world applications. This research makes both modeling and algorithmic contributions.

1. Modeling Contribution:
  • Introduces a comprehensive mixed-integer bi-level optimization model for optimal coordination between transmission and distribution networks.
  • Simultaneously models ESS at both network levels while considering demand-side management, electric vehicles, and a variety of energy resources (renewable and fossil fuels).
  • Offers a realistic representation of integrated transmission and distribution system operations.
2. Algorithm Contribution:
  • Reformulation and Decomposition Techniques: Develops an advanced algorithm that reformulates the complex bi-level model into more tractable sub-problems, improving efficiency.
  • Global Optimal Solutions: Ensures globally optimal solutions, enhancing the accuracy and reliability of the model outcomes.
  • Higher Solution Speed: Optimizes computational performance, reducing the time required for decision-making and analysis.
  • Reduced Iterations: Achieves optimal solutions with fewer iterations, lowering computational overhead and improving efficiency.
  • Computational Efficiency: The algorithm minimizes computational burden, making it suitable for large-scale problems and real-time applications.

**Fig. 1:**
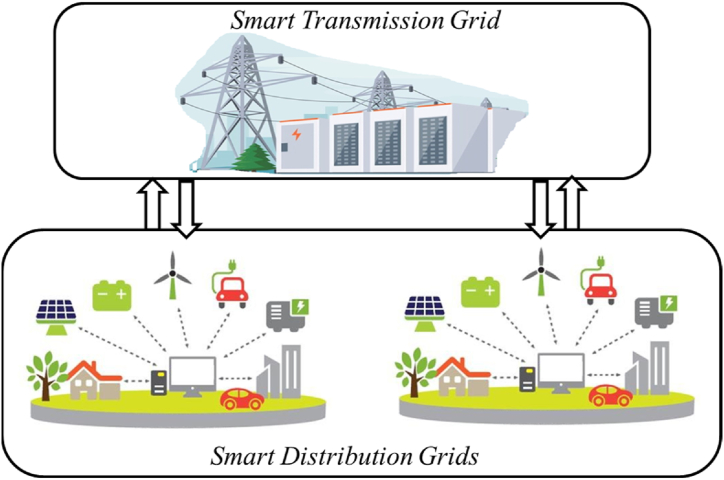
Graphical Abstract of the proposed approach.

By addressing critical gaps and introducing novel approaches in modeling and algorithm design, this research offers valuable insights for improving the coordination of transmission and distribution networks. Figure (1) illustrates the interaction between smart transmission networks, large storage systems, and smart distribution networks with renewable resources, batteries, and electric vehicles.

## 2 Problem statement

**Fig. 2:**
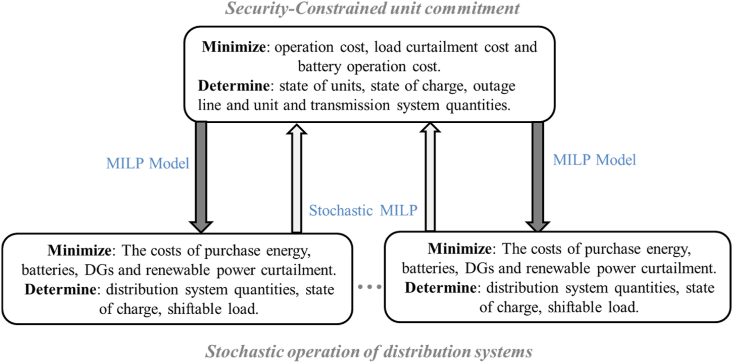
Coordinated framework of transmission and distribution network.

In this section, we present the bi-level optimization model proposed to address the coordinated operation of the transmission and distribution networks. The challenging bi-level problem is solved by considering the transmission network model as the upper-level problem and the distribution network model as the lower-level problem. The crucial link variable connecting these two networks in the proposed bi-level model is the real power that needs to be supplied from the distribution substation by the transmission network. To introduce the proposed bi-level model, we first present the transmission network model, followed by the distribution network model. Finally, we provide the solution approach to solve the proposed problem. Figure (2) illustrates the proposed framework for the coordination of the integrated transmission and distribution networks. The transmission network model captures the upper-level problem and incorporates factors such as generation scheduling, transmission line constraints, and overall system reliability. The objective is to optimize the operation of the transmission network while considering the constraints and objectives of the lower-level distribution network. The distribution network model represents the lower-level problem and takes into account factors such as load demand, renewable energy generation, energy storage systems, and distribution line constraints. The objective is to optimize the operation of the distribution network while coordinating with the upper-level transmission network. The proposed bi-level model establishes the interdependence and coordination between these two networks, enabling the determination of optimal power flow and resource allocation across the integrated system. By presenting this framework and the corresponding bi-level model, this research provides a comprehensive approach to address the coordinated operation of transmission and distribution networks, ensuring efficient and optimized system performance.

### 2.1 Upper-level model

In this paper, the upper-level problem focuses on modeling the security-constrained unit commitment (SCUC) in the transmission network. The objective function and relevant constraints of the SCUC problem are formulated as equations (1), (2), (3), (4), (5), (6), (7), (8), (9), (10), (11), (12), (13), (14), (15), (16), (17). Equation (1) represents the objective function of the upper-level problem. The specific form and details of equations (1), (2), (3), (4), (5), (6), (7), (8), (9), (10), (11), (12), (13), (14), (15), (16), (17) are not provided in the given context. However, the objective function in equation (1) captures the optimization goal of the upper-level problem, which may involve minimizing costs, maximizing system reliability, or achieving a trade-off between multiple objectives. The subsequent equations (2), (3), (4), (5), (6), (7), (8), (9), (10), (11), (12), (13), (14), (15), (16), (17) define the constraints that must be satisfied in the SCUC problem. These constraints can include factors such as power generation limits, reserve requirements, transmission line capacity, ramping constraints, and system stability criteria, among others. Each equation contributes to modeling a specific constraint to ensure the secure and reliable operation of the transmission network. By formulating the upper-level problem through equations (1), (2), (3), (4), (5), (6), (7), (8), (9), (10), (11), (12), (13), (14), (15), (16), (17), this paper provides a mathematical representation that captures the essential aspects of the security-constrained unit commitment problem in the transmission network. The subsequent sections of the paper are likely to present the lower-level problem and the integrated bi-level optimization model to address the coordinated operation of the transmission and distribution networks.

(1) $$\min\sum_{t=1}^{T}\sum_{n=1}^{N}c_{n}^{g}G_{n,t}+c_{n}^{c}u_{n,t}+c_{n}^{s}s_{n,t}+c_{n}^{d}v_{n,t}+c_{n}^{r}r_{n,t}+c_{n}^{ess}\left(p_{n,t}^{ch}+p_{n,t}^{dis}\right)$$

Equations (2), (3), (4), (5), (6), (7), (8), (9), (10), (11), (12), (13), (14), (15), (16), (17) in the paper represent various constraints related to the security-constrained unit commitment problem in the transmission network. Among these constraints, equation (2) specifically addresses the power balance limitation in the transmission network. Equation (2) is formulated as an equality constraint and ensures that the total power generation in the transmission network matches the total power demand. It captures the fundamental requirement of power balance, where the sum of power generated by all units should be equal to the total power consumed by loads and losses within the transmission network. The equation may include terms representing the power output of individual generators, power demand at different nodes, and losses in the transmission system. The equality constraint helps maintain grid stability and ensures that the power supply matches the demand.

(2) $$G_{n,t}+r_{n,t}+\sum_{n,m\in N}f_{nm,t}-\sum_{m,n\in N}f_{mn,t}-D_{n,t}-P_{n,t}^{s}+p_{n,t}^{dis}-p_{n,t}^{ch}=0$$

In relation (2), $P_{n,t}^{s}$ is equal to the power of the substation of the distribution network in the *n*th bus of the transmission network at time *t*. This power is also a common variable between two upper and lower levels. Equation (3) represents the limit of unit power in the nth bus at time t, between the minimum ($\underline{G_{n}}$) and the maximum power ($\bar{G_{n}}$) that can be produced [11]. Additionally, $z_{n,t}$ is a binary variable representing the availability of the unit in node n at time t, with a value of zero if the unit is not available, and one otherwise. Inequality (4) represents the limitation of curtailment load in the *n*th bus at time *t*. Equation (5) represents the power flow in the *nm* line, which should be considered smaller than the maximum value ($\bar{f_{nm}}$) [34].

$x_{nm}$ is equal to the susceptance between the line *nm* and $\theta_{n,t}$ is equal to the voltage angle in the *n*th bus at time *t*, and ($z_{nm,t}$) is equal to the binary variable in the security constraint limit, which is equal to zero if the *nm* line is not available at time *t*, otherwise, it is one. Equation (6) shows the logical relationship between binary variables in the problem of participation of units, if the units are clear, the binary variable $u_{n,t}$ will be equal to one. Likewise, if the units are turned off, the binary variable $v_{n,t}$ becomes equal to one, and when they are turned on again, the binary variable $s_{n,t}$ becomes equal to one. Equation (7) shows the limitation of the ramp rate of the units in the *n*th bus at time *t*. Equations (8), (9) respectively indicate the minimum up and down time of the units in the *n*th bus at time *t*. Equation (10) illustrates the limit of bus voltage angle at time *t* and equation (11) demonstrates the reference bus voltage angle. Equation (12) is related to the outage of production units and transmission lines, where (k) is the number of line and unit outages in the transmission network at time *t*. Equations (13), (14) in the paper represent the constraints related to the limitations of the battery discharge and charge power, respectively, in the coordinated operation of the transmission and distribution networks. Equation (13) sets the maximum allowable discharge power for the battery. It ensures that the rate at which the battery can discharge power does not exceed a predefined limit, considering factors such as the battery's technical specifications and operational constraints. Similarly, equation (14) establishes the maximum charge power constraint for the battery. It restricts the rate at which the battery can accept power during charging, ensuring that it does not exceed a certain threshold set by system requirements and battery characteristics. Furthermore, equation (15) represents the battery energy equation, which governs the energy dynamics of the battery over time. It describes how the battery's state of charge evolves based on the power flow into and out of the battery, considering charging efficiency and energy losses. Equation (16) represents the maximum capacity constraint of the battery. It ensures that the state of charge of the battery remains within its physical capacity limits, preventing overcharging or over-discharging, which can degrade battery performance or lead to operational issues. Lastly, equation (17) specifies the limit on the number of discharge times for the battery. This constraint restricts the frequency or frequency distribution of battery discharges, imposing a limit on the wear and tear of the battery and promoting its longevity. These equations collectively define the operational boundaries and constraints of the energy storage system, specifically the battery, in the coordinated operation of the transmission and distribution networks. They play a crucial role in optimizing the utilization and performance of the energy storage system while adhering to operational limitations and system requirements [11].

(3) $$z_{n,t}\underline{G_{n}}u_{n,t}\leq G_{n,t}\leq u_{n,t}\bar{G_{n}}z_{n,t}\;{z_{n,t},u}_{n,t}\in \left\{0,1\right\},\forall n\in N,t\in T$$

(4) $$0\leq r_{n,t}\leq D_{n,t}\;\forall n\in N,t\in T$$

(5) $$\left|f_{nm,t}={z_{nm,t}x}_{nm}\left(\theta_{n,t}-\theta_{m,t}\right)\right|\leq \bar{f_{nm}}\;\forall nm\in L,n\in N,t\in T$$

(6) $$u_{n,t+1}-u_{n,t}=s_{n,t+1}-v_{n,t+1}\;u_{n,t},s_{n,t},v_{n,t}\in \left\{0,1\right\},\forall n\in N,t\in T$$

(7) $$G_{n,t+1}-G_{n,t}\leq \bar{R_{n}},{\;G}_{n,t}-G_{n,t+1}\leq \underline{R_{n}},\forall n\in N,t\in T$$

(8) $$\sum_{k=t-{UT}_{n}+1}^{t}s_{n,k}\leq u_{n,t}\;\forall n\in N,t\in \left\{{UT}_{n},\ldots ,T\right\}$$

(9) $$\sum_{k=t-{DT}_{n}+1}^{t}v_{n,k}\leq 1-u_{n,t}\;\forall n\in N,t\in \left\{{DT}_{n},\ldots ,T\right\}$$

(10) $$\begin{array}{cc} {-\pi \leq \theta }_{n,t}\leq \pi & \forall n\in N,t\in T \end{array}$$

(11) $$\begin{array}{cc} \theta_{n,t}=0 & n=ref,t\in T \end{array}$$

(12) $$\begin{array}{cc} \sum_{n\in N}\left(1-z_{n,t}\right)+\sum_{n,m\in L}\left(1-z_{nm,t}\right)=k & \;\forall t\in T \end{array}$$

(13) $$0\leq p_{n,t}^{dis}\leq \zeta_{n,t}^{ess}X_{n}^{ess}\;\forall n\in N,t\in T$$

(14) $$0\leq p_{n,t}^{ch}\leq \left(1-\zeta_{n,t}^{ess}\right)X_{n}^{ess}\;\forall n\in N,t\in T$$

(15) $$e_{n,t}^{ess}=e_{n,t-1}^{ess}+\eta_{n}^{ess}p_{n,t}^{ch}-\frac{p_{n,t}^{dis}}{\eta_{n}^{ess}}\;\forall n\in N,t\in T$$

(16) $$0\leq e_{n,t}^{ess}\leq X_{n}^{ess}\;\forall n\in N,t\in T$$

(17) $$\sum_{t\in T}\zeta_{n,t}^{ess}=Α\;\forall n\in N$$

As seen, the upper-level problem (1), (2), (3), (4), (5), (6), (7), (8), (9), (10), (11), (12), (13), (14), (15), (16), (17) is a mixed integer linear programming problem, which can be solved by powerful commercial solvers. In the next section, the lower-level problem related to the distribution network is presented.

### 2.2 Lower-level model

In this section of the paper, the optimization problem for the distribution network is presented as a mixed integer linear model, represented by equations (18), (19), (20), (21), (22), (23), (24), (25), (26), (27), (28), (29), (30), (31), (32), (33), (34), (35), (36), (37), (38), (39), (40). Equation (18) corresponds to the objective function of the lower-level problem. Equation (18) represents the objective function of the distribution network optimization problem. It defines the goal to be minimized or maximized in the context of the coordinated operation of the transmission and distribution networks. The specific form of the objective function depends on the objectives and criteria considered in the study, such as minimizing power losses, maximizing renewable energy utilization, or minimizing operational costs. The objective function takes into account various components and variables that affect the performance and efficiency of the distribution network. It could include terms related to power flow, voltage regulation, load balancing, and other factors relevant to the optimization objectives. By formulating the objective function, researchers aim to find the optimal values for decision variables and system parameters that achieve the desired optimization goals for the distribution network. Equations (19), (20), (21), (22), (23), (24), (25), (26), (27), (28), (29), (30), (31), (32), (33), (34), (35), (36), (37), (38), (39), (40) denote the constraints associated with the distribution network optimization problem. These constraints consider technical and operational aspects of the distribution system, such as power flow equations, voltage limits, load demand, renewable energy generation, and operational constraints. Each equation represents a specific constraint that must be satisfied to ensure the feasibility and reliability of the distribution network's operation. Together, equations (18), (19), (20), (21), (22), (23), (24), (25), (26), (27), (28), (29), (30), (31), (32), (33), (34), (35), (36), (37), (38), (39), (40) constitute a comprehensive mathematical representation of the distribution network optimization problem within the context of the coordinated operation with the transmission network. The objective function and constraints provide a framework for finding the optimal solution that balances various objectives and constraints in the distribution network.

(18) $$\min\sum_{t=1,n=ref}^{T}c_{t}^{s}\left(P_{n,t}^{s}+Q_{n,t}^{s}\right)+\sum_{n=1}^{N}\sum_{t=1}^{T}c_{n}^{DG}\left(P_{n,t}^{DG}\right)+\sum_{n=1}^{N}\sum_{t=1}^{T}c_{n}^{ess}\left(p_{n,t}^{ch}+p_{n,t}^{dis}\right)+\sum_{n,m\in N}\sum_{t=1}^{T}c_{t}^{loss}\left(f_{nm,t}^{p}+f_{mn,t}^{p}\right)+\sum_{s=1}^{S}\sigma_{s}\left(\sum_{n=1}^{N}\sum_{t=1}^{T}c_{n}^{RN}\left({\bar{P}}_{n,t,s}^{RN}-P_{n,t}^{RN}\right)+c_{n}^{PEV}\left({\bar{P}}_{n,t,s}^{PEV}-P_{n,t}^{PEV}\right)\right)$$

In relation (18), the first objective is to minimize the cost of real power ($P_{n,t}^{s}$) and reactive power ($Q_{n,t}^{s}$) purchased from the transmission network at time t at the distribution substation. The second objective is to reduce the production cost of DG and the third objective is to reduce the cost of charging and discharging the battery. The fourth objective is to reduce the cost of the distribution network line power losses. In this paper, power losses, are computed as the sum of direct and reverse line flows [11]. The final objective is to minimize the cost of non-participation of renewable resources or electric vehicle charging stations, ensuring that the maximum power of renewable resources is utilized every hour at every bus of the distribution network and the charging power of electric vehicles is supplied every hour at every bus. Equations (19), (20) represent the constraints for active and reactive power balance in the distribution network.

(19) $$P_{n=ref,t}^{s}+\sum_{n,m\in N}f_{nm,t}^{p}-\sum_{m,n\in N}f_{mn,t}^{p}+P_{n,t}^{RN}-P_{n,t}^{PEV}+P_{n,t}^{DG}-{\tilde{d}}_{n,t}^{p}+p_{n,t}^{dis}-p_{n,t}^{ch}=0\;\forall n\in N,t\in T$$

(20) $$Q_{n=ref,t}^{s}+\sum_{n,m\in N}f_{nm,t}^{q}-\sum_{m,n\in N}f_{mn,t}^{q}+{\rho_{n}P}_{n,t}^{DG}-{\tilde{d}}_{n,t}^{q}=0\;\forall n\in N,t\in T$$

In relation (19), $P_{n=ref,t}^{s}$ is equal to the real power of the distribution substation at time *t*. Similarly, in relation (20), $Q_{n=ref,t}^{s}$ is equal to the reactive power of the distribution substation at time *t*. Equations (21), (22), (23) indicate the limited use of renewable resources, DG production, and electric vehicle charging stations, respectively [32].

(21) $$0\leq P_{n,t}^{PV}\leq {\bar{P}}_{n,t,s}^{PV}\;\forall n\in N,t\in T,s\in S$$

(22) $$0\leq P_{n,t}^{DG}\leq {\bar{P}}_{n,t}^{DG}\;\forall n\in N,t\in T$$

(23) $$0\leq P_{n,t}^{PEV}\leq {\bar{P}}_{n,t,s}^{PEV}\;\forall n\in N,t\in T,s\in S$$

Equations (24), (25) show the limits of real and reactive power flow between *nm* lines at time *t*, respectively.

(24) $${\underline{f}}_{nm}^{p}\leq f_{nm,t}^{p}\leq {\bar{f}}_{nm}^{p}\;\forall nm\in L,t\in T$$

(25) $${\underline{f}}_{nm}^{q}\leq f_{nm,t}^{q}\leq {\bar{f}}_{nm}^{q}\;\forall nm\in L,t\in T$$

In relations (26) and (27) the maximum active (${\bar{P}}_{n}^{s}$) and reactive (${\bar{Q}}_{n}^{s}$) power of the distribution substation at time *t* is shown.

(26) $$P_{n,t}^{s}\leq {\bar{P}}_{n}^{s}\;n=ref,t\in T$$

(27) $$Q_{n,t}^{s}\leq {\bar{Q}}_{n}^{s}\;n=ref,t\in T$$

Equation (28) shows the square voltage of the *n*th bus of the distribution network at time *t*. Inequality (29) demonstrates the bus voltage limit of the distribution network. Here we have considered the voltage between 0.9^2^ and 1.1^2^ p.u [11].

(28) $$V_{n,t}^{sqe}=V_{m,t}^{sqe}-2\left(r_{nm}f_{nm,t}^{p}-x_{nm}f_{nm,t}^{q}\right)\;\forall n\in N,nm\in L,t\in T$$

(29) $$V_{n}^{\min}\leq V_{n,t}^{sqe}\leq V_{n}^{\max}\;n\in N,t\in T$$

Equations (30), (31), (32), (33), (34) illustrate the battery modeling in the distribution network. Like the battery in the transmission network, here also the relations (30) and (31) demonstrate the limitation of discharging and charging the battery, respectively. Equation (32) illustrates the battery energy and equation (33) shows the battery energy limit. Finally, equation (34) indicates the limitation of the number of battery discharge times [34].

(30) $$0\leq p_{n,t}^{dis}\leq \zeta_{n,t}^{ess}X_{n}^{ess}\;\forall n\in N,t\in T$$

(31) $$0\leq p_{n,t}^{ch}\leq \left(1-\zeta_{n,t}^{ess}\right)X_{n}^{ess}\;\forall n\in N,t\in T$$

(32) $$e_{n,t}^{ess}=e_{n,t-1}^{ess}+\eta_{n}^{ess}p_{n,t}^{ch}-\frac{p_{n,t}^{dis}}{\eta_{n}^{ess}}\;\forall n\in N,t\in T$$

(33) $$0\leq e_{n,t}^{ess}\leq X_{n}^{ess}\;\forall n\in N,t\in T$$

(34) $$\sum_{t\in T}\zeta_{n,t}^{ess}=Α\;\forall n\in N$$

The demand-side management model presented in the paper is represented by equations (35), (36), (37), (38), (39), (40). These equations define the constraints and objectives related to the management of electricity demand within the coordinated operation of the transmission and distribution networks. Equations (35), (38) ensure that no active and reactive load shedding occurs in the demand side management plan. Load shedding refers to the intentional reduction of electrical load in response to system constraints or grid stability concerns. These equations set the conditions to avoid load shedding and maintain the reliability of the power supply. Equations (36), (37) impose limits on the magnitude of active load changes. These equations indicate the maximum allowable increase or decrease in the active power demand during the demand side management program. By specifying these limits, the model ensures that the load changes are within acceptable bounds and do not cause disruptions or instability in the distribution network. Similarly, equations (39), (40) establish limits on the magnitude of reactive load changes. These equations control the variation in reactive power demand, which is related to voltage control and stability. By setting boundaries on reactive load changes, the model ensures that the demand side management program operates within the defined limits to maintain appropriate voltage levels and system performance. Overall, equations (35), (36), (37), (38), (39), (40) in the demand-side management model capture the constraints and limitations related to load shedding, active load changes, and reactive load changes. These constraints are essential for optimizing the demand side management program and achieving the desired objectives of the coordinated operation of the transmission and distribution networks [32].

(35) $$\sum_{t\in T}d_{n,t}^{p}=\sum_{t\in T}{\tilde{d}}_{n,t}^{p}\;\forall n\in N$$

(36) $$\begin{array}{cc} {\tilde{d}}_{n,t}^{p}\leq d_{n,t}^{p}+d_{n,t}^{p}\varepsilon & \forall n\in N,t\in T \end{array}$$

(37) $$\begin{array}{cc} {\tilde{d}}_{n,t}^{p}\geq d_{n,t}^{p}-d_{n,t}^{p}\varepsilon & \forall n\in N,t\in T \end{array}$$

(38) $$\sum_{t\in T}d_{n,t}^{q}=\sum_{t\in T}{\tilde{d}}_{n,t}^{q}\;\forall n\in N$$

(39) $$\begin{array}{cc} {\tilde{d}}_{n,t}^{q}\leq d_{n,t}^{q}+d_{n,t}^{q}\varepsilon & \forall n\in N,t\in T \end{array}$$

(40) $$\begin{array}{cc} {\tilde{d}}_{n,t}^{q}\geq d_{n,t}^{q}-d_{n,t}^{q}\varepsilon & \forall n\in N,t\in T \end{array}$$

In the next section of the paper, the authors describe the method used to solve the proposed bi-level optimization problem, which involves addressing the challenges posed by the presence of binary variables at both the upper-level and lower-level models. To solve the problem, an exact approach based on decomposition algorithms is proposed. This approach aims to decompose the bi-level model into two separate optimization problems: one for the upper-level (transmission network) and one for the lower-level (distribution network). By decomposing the problem, it becomes more manageable and solvable. The authors utilize reformulation and decomposition techniques to handle the binary variables in the model and ensure the generation of global optimal solutions. These techniques help in transforming the original problem into smaller sub-problems that can be solved individually, and then combining the solutions to obtain an optimal solution for the entire problem. The exact approach presented in the paper guarantees the optimality of the solutions obtained. It also offers advantages in terms of solution speed, requiring fewer iterations compared to other methods. Additionally, the proposed model and algorithm have the capability to integrate any number of distribution networks into the transmission network, allowing for scalability and adaptability to different system configurations. By providing a detailed methodology for solving the proposed bi-level optimization problem, the authors contribute to the understanding and practical implementation of coordinated operation strategies for integrated transmission and distribution networks with energy storage systems.

## 3 Proposed algorithm

As elucidated in the preceding section, the upper-level and lower-level models can both be expressed as formulations of mixed integer linear programming. Essentially, equations (41), (42), (43), (44) encapsulate the fundamental framework of the proposed bi-level problem.

(41) $$\text{Upper}\;\text{Level}=\min\sum_{t=1}^{T}\sum_{n=1}^{N}c_{n}^{g}G_{n,t}+c_{n}^{c}u_{n,t}+c_{n}^{s}s_{n,t}+c_{n}^{d}v_{n,t}+c_{n}^{r}r_{n,t}+c_{n}^{ess}\left(p_{n,t}^{ch}+p_{n,t}^{dis}\right)$$

(42) s.t(2)−(17)

(43) $$\text{Lower}\;\text{Level}=\min\;\omega_{1}\sum_{t=1,n=ref}^{T}c_{t}^{s}\left(P_{n,t}^{s}+Q_{n,t}^{s}\right)+\omega_{2}\sum_{n=1}^{N}\sum_{t=1}^{T}c_{n}^{DG}\left(P_{n,t}^{DG}\right)+\omega_{3}\sum_{n=1}^{N}\sum_{t=1}^{T}c_{n}^{ess}\left(p_{n,t}^{ch}+p_{n,t}^{dis}\right)+\omega_{4}\sum_{n,m\in N}\sum_{t=1}^{T}c_{t}^{loss}\left(f_{nm,t}^{p}+f_{mn,t}^{p}\right)+\omega_{5}\sum_{s=1}^{S}\sigma_{s}\left(\sum_{n=1}^{N}\sum_{t=1}^{T}c_{n}^{RN}\left({\bar{P}}_{n,t,s}^{RN}-P_{n,t}^{RN}\right)+c_{n}^{PEV}\left({\bar{P}}_{n,t,s}^{PEV}-P_{n,t}^{PEV}\right)\right)$$

(44) s.t(19)−(40)

To tackle the difficulties arising from the binary variables in the upper and lower levels of the proposed bi-level model, we utilize a precise methodology inspired by the approach outlined in references [36,37]. In particular, we employ a reformulation-and-decomposition framework that incorporates techniques of column-and-constraint generation. This approach entails iteratively solving a master problem and two subproblems to compute equations (45), (46), (47), (48), (49), (50), (51), (52). To demonstrate the sequential steps of our computational methodology, we present a succinct depiction of equations (45), (46), (47), (48), (49), (50), (51), (52) as follows:

(45) minax+by

(46) $$s.t.\;By+Cr_{n}=d$$

(47) Ax+Dy≥l,xϵ{0,1}

(48) ‖Hy‖≤hy

(49) $$\text{where}\;r_{n}\in \mathrm{argmin}\{e_{n}\left(r_{n}+y_{n}\right)\text{:}$$

(50) $$s.t.\;{\;G}_{n}w_{n}+E_{n}r_{n}+O_{n}y_{n}=f_{n}$$

(51) $$F_{n}w_{n}+K_{n}r_{n}\leq k_{n}$$

(52) $$r_{n}\geq 0,w_{n}\in \left\{0,1\right\}\}\;\forall n\in N$$

In the given context, the variables x and y represent the binary and continuous variables at the upper level, while the lower-level continuous and binary variables associated with the independent smart distribution grid connected to transmission node n are denoted by $r_{n}$ and $w_{n}$, respectively.

The variables are characterized by coefficient matrices and vectors ($A,a,B,b,E_{n},e_{n},l,D,d,F_{n},f_{n},G_{n},H,h,K_{n},k_{n},O_{n}$) with appropriate dimensions. Inequality (48) employs the symbol ‖·‖ to denote the $l_{2}$-norm for matrices and vectors. Equation (49) defines the objective function, while equations (50), (51), (52) represent the primal constraints of the lower-level model, specifically pertaining to the independent smart distribution grid connected to transmission node n.

To facilitate the development of the algorithm, it is necessary to establish a decomposable structure. In this investigation, we have employed the methodology outlined in Ref. [41] to restructure the bi-level model expressed by equations (45), (46), (47), (48), (49), (50), (51), (52). This reformulation entails replicating the lower-level variables and constraints within the upper-level model while introducing an additional constraint (equation (57)) as demonstrated below:

(53) minax+by

(54) s.t.[(46)−(48)]

(55) $$\begin{array}{cc} G_{n}w_{n}^{\prime }+E_{n}r_{n}^{\prime }+O_{n}y_{n}=f_{n}\text{,} & F_{n}w_{n}^{\prime }+K_{n}r_{n}^{\prime }\leq k_{n} \end{array}$$

(56) $$r_{n}^{\prime }\geq 0,w_{n}^{\prime }\in \left\{0,1\right\}$$

(57) $$e_{n}\left(r_{n}^{\prime }+y_{n}\right)\leq \min\;\{e_{n}\left(r_{n}+y_{n}\right)\text{:}$$

(58) $$\begin{array}{cc} s.t.\;{\;G}_{n}w_{n}+E_{n}r_{n}+O_{n}y_{n}=f_{n}\text{,} & F_{n}w_{n}+K_{n}r_{n}\leq k_{n} \end{array}$$

(59) $$\begin{array}{ccc} r_{n}\geq 0\text{,} & w_{n}\in \left\{0,1\}\right\} & \forall n\in N \end{array}$$

In the upper-level, the lower-level variables that are duplicated are represented by $r_{n}^{\prime }$ and $w_{n}^{\prime }$. It is important to note that equations (55), (56) specifically apply to the independent smart distribution grid. With the inclusion of equation (57), we establish the equivalence of the model (53)–(59) with the primary optimization problem of the bi-level model (1)–(40). Although more intricate than (1)–(40), the reformulated problem (53), (54), (55), (56), (57), (58), (59) provides a useful framework for deriving non-trivial bounds for problem (1), (2), (3), (4), (5), (6), (7), (8), (9), (10), (11), (12), (13), (14), (15), (16), (17), (18), (19), (20), (21), (22), (23), (24), (25), (26), (27), (28), (29), (30), (31), (32), (33), (34), (35), (36), (37), (38), (39), (40). Let W denote the set of all feasible realizations of $w_{n}$, and let ${\tilde{w}}_{n}$ be a representative realization of $w_{n}$. By exhaustively enumerating $w_{n}$ and presenting its continuous variables ${\hat{r}}_{n}^{n}$, we can express equations (57), (58), (59) as follows:

(60) $$e_{n}\left(r_{n}^{\prime }+y_{n}\right)\leq \max\{e_{n}\left({\hat{r}}_{n}^{{\tilde{w}}_{n}}+y_{n}\right)$$

(61) $$s.t.\;G_{n}{\hat{r}}_{n}^{{\tilde{w}}_{n}}+E_{n}{\tilde{w}}_{n}+O_{n}y_{n}=f_{n}$$

(62) $$K_{n}{\hat{r}}_{n}^{{\tilde{w}}_{n}}+F_{n}{\tilde{w}}_{n}\leq k_{n}$$

(63) $${\hat{r}}_{n}^{{\tilde{w}}_{n}}\geq 0,{\tilde{w}}_{n}\in W,n\in N$$

Given a specific ${\tilde{w}}_{n}$, it is evident that the right-hand side of equations (60), (61), (62), (63) forms a linear model. Furthermore, instead of exhaustively enumerating all possibilities, equations (60), (61), (62), (63) are derived based on a subset $\tilde{W}\subseteq W$, which provides a relaxation of (53), (54), (55), (56), (57), (58), (59) or equivalently the primary bi-level problem (1), (2), (3), (4), (5), (6), (7), (8), (9), (10), (11), (12), (13), (14), (15), (16), (17), (18), (19), (20), (21), (22), (23), (24), (25), (26), (27), (28), (29), (30), (31), (32), (33), (34), (35), (36), (37), (38), (39), (40). As detailed in the subsequent section, these characteristics allow us to employ the column-and-constraint generation method [41] for the development of the decomposition technique.

### 3.1 Subproblems

For a given upper-level decision ($x^{*},y^{*}$), the subsequent subproblem SP1_n_ is formulated and computed for a gas-fired unit connected to gas node n∈N.

(64) $${SP1}_{n}:\Psi_{n}\left(x^{*},y_{n}^{*}\right)=\min\;e_{n}\left(r_{n}+y_{n}^{*}\right)$$

(65) $$s.t.\;G_{n}w_{n}+E_{n}r_{n}+O_{n}y_{n}^{*}=f_{n}:\lambda_{n}$$

(66) $$F_{n}w_{n}+K_{n}r_{n}\leq k_{n}:\mu_{n}$$

(67) $$r_{n}\geq 0,w_{n}\in W$$

Although the problem SP1_n_ involves mixed-integer linear optimization, it is worth noting that for a fixed $w_{n}$, the remaining portion of the problem is linear. The dual variables associated with constraints (65) and (66) are represented by $\lambda_{n}$ and $\mu_{n}$, respectively. Specifically, SP1n provides an optimal solution for the lower-level (43)–(44) with respect to the investment plan ($x^{*},y_{n}^{*}$). However, it is important to acknowledge that there may exist multiple solutions. The second subproblem, denoted as SP2, is derived from an upper-level problem and is formulated as a mixed-integer model, presented below:

(68) $$SP2:\Psi \left(x^{*},y^{*}\right)=\min\;ax+by$$

(69) s.t.[(65)−(67)]

(70) $$e_{n}\left(r_{n}+y_{n}\right)\leq \Psi_{n}\left(x^{*},y_{n}^{*}\right)\;\forall n\in N$$

### 3.2 Master problem

By employing the formulations (53)–(63), the creation of the master problem involves two key steps.

1) Duplicating the lower-level variables (indicated as $w_{n}^{\prime }$ and $r_{n}^{\prime }$) and constraints within the upper-level model.
2) Replacing the lower-level model (at iteration n) with a fixed realization ${\tilde{w}}_{n}^{j}\in \tilde{W}\subseteq W$ by its Karush–Kuhn–Tucker conditions. The continuous primal variables are denoted as ${\hat{r}}_{n}^{j}$, and the dual variables are denoted as $\lambda_{n}^{j}$ and $\mu_{n}^{j}$.

The algorithm description suggests the dense form of the master problem (represented by equations (71)–(76) below and also documented in Ref. [41]). The master problem (MP) formulated for our study encapsulates equations (71)–(76), designed to optimize the specified objectives under defined constraints. Equation (71) sets the objective function Π to be minimized, expressed in terms of variables ax and by. This objective function aims to achieve optimal values that align with our optimization goals. Equation (72) encompass a series of constraints, incorporating [(45)–(48)] and [(55)–(56)], applicable universally across the set N. These constraints ensure that the optimization process respects all necessary conditions and requirements. Equation (73) introduces constraints involving terms such as $G_{n}{\tilde{w}}_{n}^{j}$, $E_{n}{\hat{r}}_{n}^{j}$, $O_{n}$, and $y_{n}$, where 1≤j≤τ and n∈N. These terms play crucial roles in defining relationships and limitations within the optimization framework. Equation (74) presents an inequality constraint that stipulates the relationship between $e_{n}$, $r_{n}^{\prime }+y_{n}$, and ${\hat{r}}_{n}^{j}+y_{n}$ for 1≤j≤τ and n∈N. This inequality ensures that certain conditions are met, contributing to the feasibility and robustness of the optimization model. Equation (75) introduces non-negativity and feasibility constraints involving $\mu_{n}^{j}$, $F_{n}$, ${\tilde{w}}_{n}^{j}$, $K_{n}$, ${\hat{r}}_{n}^{j}$, and $k_{n}$, for 1≤j≤τ and n∈N. These constraints are essential for maintaining the integrity and practicality of the optimization solution. Equation (76) outlines additional constraints pertaining to ${\hat{r}}_{n}^{j}$, $E_{n}$, $\lambda_{n}^{j}$, $K_{n}$, $\mu_{n}^{j}$, $e_{n}$, and their interdependencies for 1≤j≤τ and n∈N. These constraints further refine the optimization model, ensuring that all relevant variables and relationships are accounted for in the solution process. Together, equations (71)–(76) form the foundational elements of our optimization approach, providing a structured framework to address the complexities and requirements of [specific problem domain or application]. These equations are integral to achieving optimal solutions that meet our defined objectives effectively.

The following outlines the steps of the adapted column-and-constraint generation decomposition method proposed for solving the comprehensive bi-level optimization model. In this context, LB represents the lower bounds, UB denotes the upper bounds, ϵ signifies the optimality tolerance, and ς represents the iteration index.

The adapted column-and-constraint generation decomposition method is specifically devised to systematically improve the lower bounds obtained from the master problem and the upper bounds obtained from the subproblems. In each iteration, the method incorporates new variables and constraints into the master problem until the discrepancy between the bounds becomes smaller than the specified optimality tolerance ϵ. A comprehensive mathematical proof regarding the algorithm's convergence to the global optimal value can be found in Ref. [42].

**Fig. 3:**
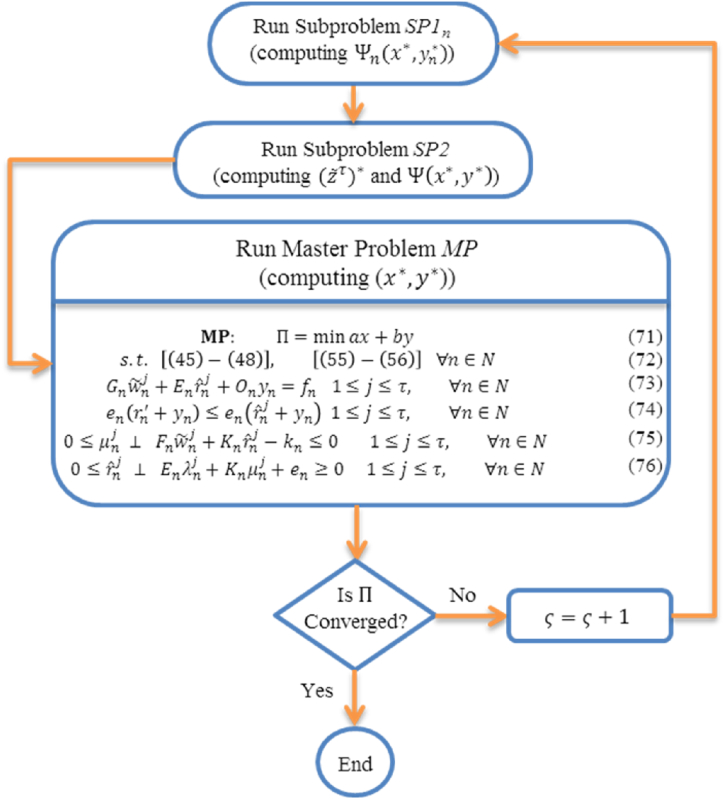
Flowchart of the proposed bi-level solution.

The flowchart depicted in Fig. 3 presents the schematic of the proposed bi-level solution approach. It is noteworthy that the master problem can be converted into a standard mixed-integer linear program by applying the big-M method to linearize equations (75) and (76). This transformation allows for the utilization of existing commercial mixed-integer solvers to effectively solve both the subproblems and the master problem.

### 3.3 Practical implications

Bi-level optimization in power transmission and distribution networks has diverse practical applications [56], encompassing market-based power dispatch, voltage control, reactive power optimization, demand response and load management, optimal power flow in distribution networks, renewable energy integration, and transmission expansion planning. It facilitates the coordination and optimization of decisions between upper-level entities such as market operators, system operators, demand response aggregators, distribution system operators, grid operators, and planners, and lower-level entities including generators, devices and controllers, consumers, distributed energy resources, renewable energy producers, and investors, resulting in enhanced efficiency, reliability, and sustainability in the overall operation of the electricity grid.

### 3.4 Limitation

While bi-level models and reformulation/decomposition algorithms provide valuable insights and solutions to hierarchical decision-making problems, their practical application is constrained by computational complexity, as solving bi-level optimization problems can be computationally expensive, particularly with a large number of decision variables and constraints, and depending on the problem structure, decomposition may not always result in a significant reduction in computational complexity. Additionally, these approaches exhibit sensitivity to model parameters, where the model's performance may be affected by the choice of parameters and formulation of upper and lower-level objectives, and the effectiveness of decomposition methods may depend on the specific characteristics of the problem being addressed. The assumption of rationality in bi-level models, positing that decision-makers at both levels are rational and possess complete information, may not always align with real-world scenarios, and the applicability of decomposition methods may be limited by the problem structure and the complexity of interactions between decision variables. Consequently, the practical use of bi-level models and decomposition algorithms requires careful consideration, accounting for the unique characteristics and limitations of the specific decision-making scenario at hand.

In the subsequent section, we will analyze and present the results obtained from the simulations.

## 4 Simulation results

This section aims to validate the proposed model and method through the examination of three distinct integrated systems: a 6-bus transmission system, a 14-bus transmission system, and a 30-bus transmission system. Moreover, the simulations encompass a comprehensive 118-bus network, alongside multiple distribution networks [11,13,32,34]. The simulations were performed on a laptop equipped with a 2.2 GHz processor and 16 GB of RAM, utilizing the Julia programming language and the Gurobi solver version 9 [43]. The number of 20 scenarios for renewable energy resources and electric vehicles is considered, which is obtained using the probability density function [34].

### 4.1 First integrated system

**Fig. 4:**
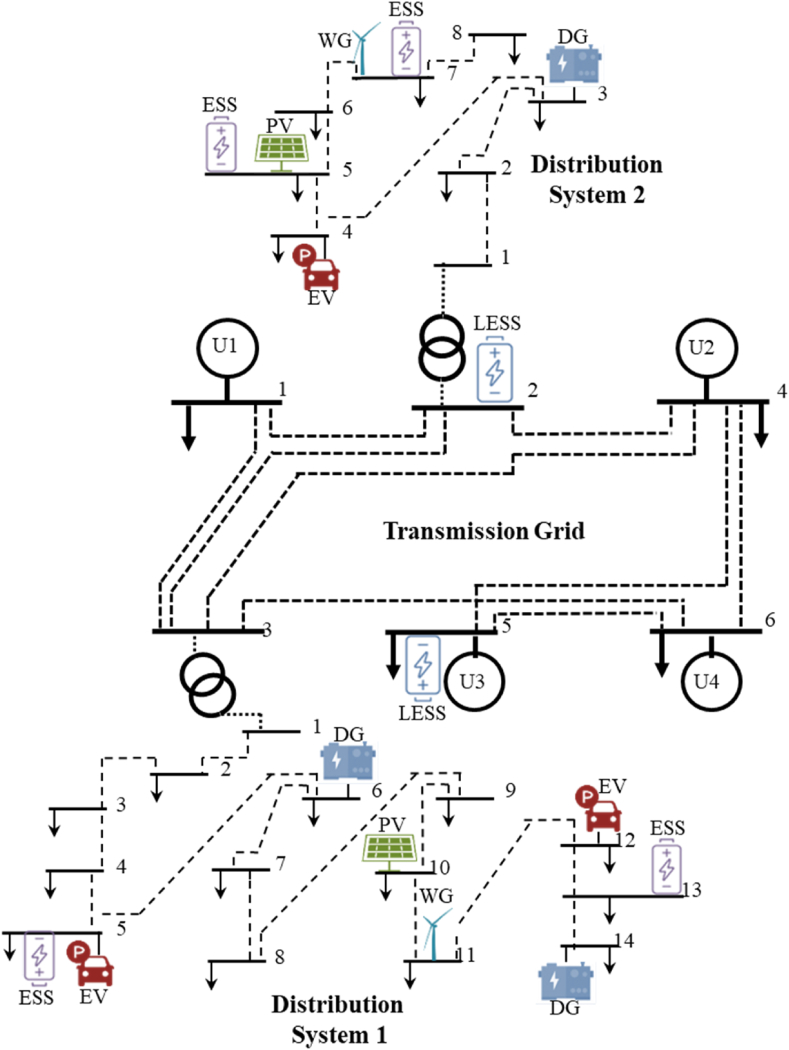
Diagram of the first proposed joint system.

Fig. 4 illustrates the configuration of the simulated transmission network, which consists of 6 buses and 9 lines [11]. Within this network, there are 4 power plant units and 2 large-scale energy storage systems, positioned at buses 2 and 5, respectively. Additionally, two distribution networks are incorporated, connected to buses 2 and 3 of the transmission network. The first distribution network encompasses 14 buses and 13 lines, featuring electric vehicle charging stations at buses 5 and 12, as well as non-renewable production facilities at buses 6 and 14.

Within the first distribution network, photovoltaic (PV) units are installed at bus 10, while wind units are situated at bus 11. Batteries are also present at buses 5 and 13. On the other hand, the second distribution network, linked to bus 2 of the transmission network, consists of 8 buses and 7 lines. It encompasses a fossil generator at bus 3, an electric vehicle charging station at bus 4, and PV and wind units at buses 5 and 7. Batteries are installed at buses 5 and 7 within this network as well. To thoroughly examine the proposed model and assess the reciprocal impacts resulting from alterations in both the transmission and distribution networks, numerous scenarios have been taken into account.

The simulation analysis consists of three cases.

Case 1 Normal Operation - This case represents the baseline scenario where all components, including batteries, renewable resources, and fossil resources, are considered at both the transmission and distribution network levels.

Case 2 Exclusion of Batteries and Resources in Distribution Network - In this case, the batteries, renewable resources, and fossil resources at the distribution network level are not taken into account. The focus is on the operation of the transmission network and its coordination with the distribution network.

Case 3 Exclusion of Battery at Transmission Network Level - Here, the battery at the transmission network level is excluded from the analysis, while other components and resources are considered in both the transmission and distribution networks.

These cases allow for the examination of different scenarios and the evaluation of the impact of specific components on the coordinated operation of the integrated transmission and distribution networks.

Case 1 represents the baseline scenario where all components, including batteries, renewable resources, and fossil resources, are considered at both the transmission and distribution network levels. This scenario aims to reflect normal operations without excluding any major energy resources, providing a comprehensive view of how the system functions under typical conditions. The results of this case show an upper objective function value of $6951 and a lower objective function value of $1928. The substation power for the first distribution network is 6.29 MW, while for the second distribution network it is 47.2 MW. The total transmission network power is 222.5 MW. Line outages occur 9 times, distributed over various times, with some lines experiencing multiple outages. There is also a unit outage occurring once at time 18.

In Case 2, batteries, renewable resources, and fossil resources at the distribution network level are excluded from the analysis. The focus shifts to the operation of the transmission network and its coordination with the distribution network, without the additional support from the distribution-level resources. The upper objective function value increases to $7092, indicating a higher cost of operation under these conditions. The lower objective function value also increases to $2204. The substation power for the first distribution network rises to 8.76 MW, and for the second distribution network to 51.5 MW. The total transmission network power increases to 231 MW. Line outages occur 8 times, with a significant distribution of outages over different times, indicating a more strained network. Unit outages occur twice at times 12 and 13.

Case 3 excludes the battery at the transmission network level while considering other components and resources in both the transmission and distribution networks. This case presents a significant deviation from the previous ones, with the upper objective function value skyrocketing to $41319, suggesting a substantial increase in operational costs when the transmission-level battery is removed. The lower objective function value remains at $1928, unchanged from Case 1. The substation power for the first distribution network is 6.3 MW, and for the second distribution network, it is 47.2 MW. The total transmission network power drops dramatically to 182.5 MW, indicating a major reduction in available power. Line outages occur 9 times, with a diverse distribution across different times, reflecting significant network instability. Unit outages occur six times, spread over various times, highlighting the challenges faced without the transmission-level battery.

The analysis of these three cases reveals how the exclusion of key components, such as batteries and distribution-level resources, impacts the overall performance and costs associated with the energy network. Case 1, with all components operational, serves as a baseline with lower costs and fewer outages. In contrast, Case 2 and Case 3 show increased costs and more frequent outages, highlighting the critical role of batteries and distribution-level resources in maintaining efficient and stable energy network operations.

**Fig. 5:**
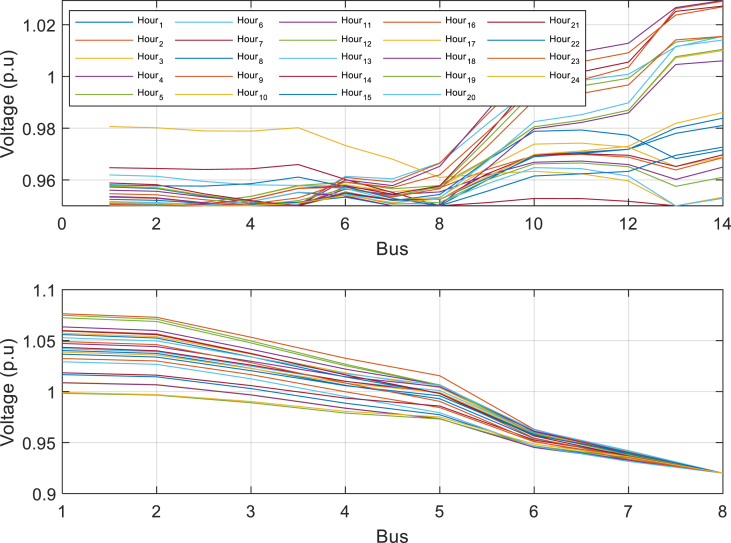
Bus voltage of distribution networks in 24 h considered in the first case.

In order to demonstrate the impact of modeling resources available in distribution networks, such as batteries and DG production resources, on the transmission network, and to validate the proposed method and model, the results of the second case are also presented. As evident from Table (2), in the second case, the removal of wind, solar, and fossil resources, as well as batteries, from the distribution networks has had a significant impact on the transmission network. The objective function of the upper problem has increased from 6951 to 7092 dollars, indicating the influence of these changes. Based on the data in Table 2, in Case 2, the objective function of the lower problem increased by approximately 141 dollars compared to Case 1. This increase in the objective function reflects the impact of resources in the distribution network. The amount of power purchased in the distribution substation over 24 h in Case 2 is 8.76 MW and 51.5 MW. In the same way, the amount of power produced by the power plant units of the transmission network increased from 223 to 231 MW. Table 2 confirms the effectiveness and performance of the proposed method and model. Fig. 5 displays the voltage of buses 14 and 8 in the distribution networks.

**Table 2:** The results of the simulation of the first system in a 24-h period.

	case 1	case 2	case 3
objective function of the upper problem ($)	6951	7092	41319
objective function of the lower problem ($)	1928	2204	1928
Substation power of the first distribution network (MW)	6.29	8.76	6.3
Substation power of the second distribution network (MW)	47.2	51.5	47.2
Total transmission network power (MW)	222.5	231	182.5
line outage (time)	9(1,11,17,21,22,24)	1(1)	9(1،11،20)
	8(2,5,9,12,15,19,20,23)	5(2)	8(2،10،13)
	7(3,4,7)	9(3،6،22)	7(3،4،12،14،16،18،19،21)
	3(6,8,10,13)	7(4،5،7،23،24)	5(6)
	6(14)	8(8،9،10،11،14–19)	6(15)
	5(16)	3(20)	1(17)
		5(21)	
unit outage (time)	2(18)	2(12،13)	2(5،7–9،22-24)

**Fig. 6:**
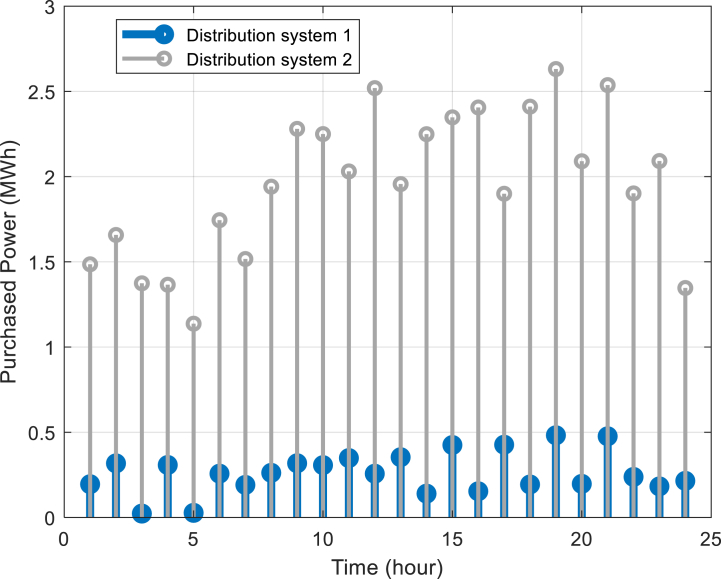
Purchased power of distribution networks in 24 h in the first case.

**Fig. 7:**
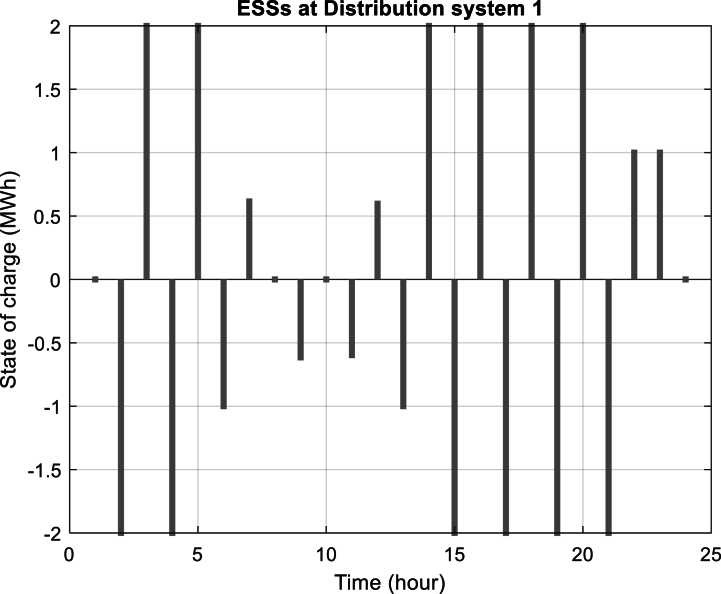
Optimum power of charging and discharging batteries in the first distribution system in the first case.

**Fig. 8:**
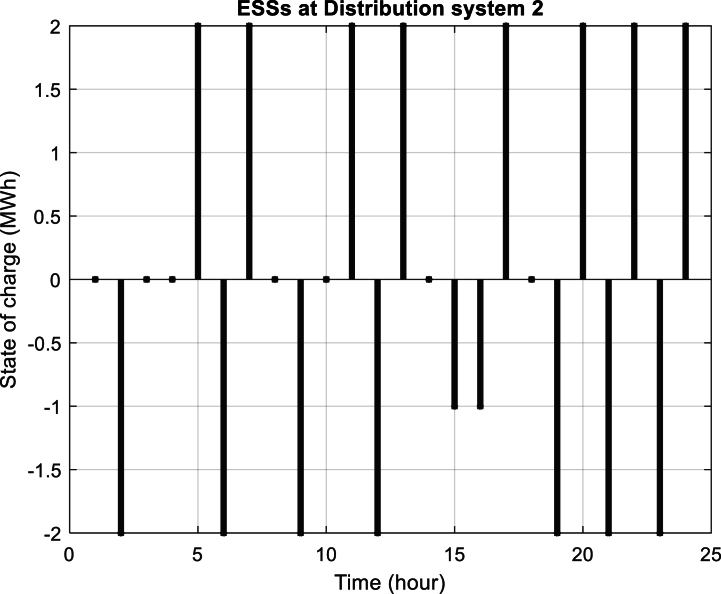
Optimum power of charging and discharging batteries in the second distribution system in the first case.

**Fig. 9:**
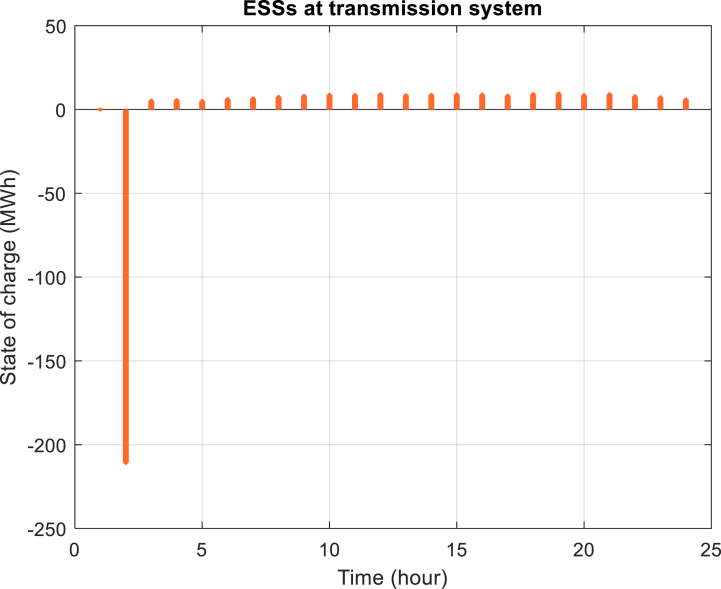
Optimal charging and discharging power of batteries in the transmission network in the first case.

**Fig. 10:**
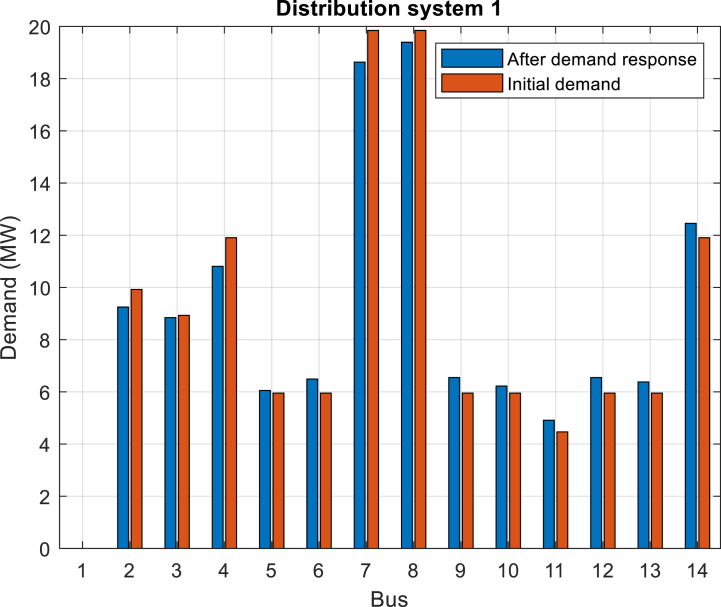
Comparison of the active load of the first distribution network in the initial state with the demand response management state in the first case.

**Fig. 11:**
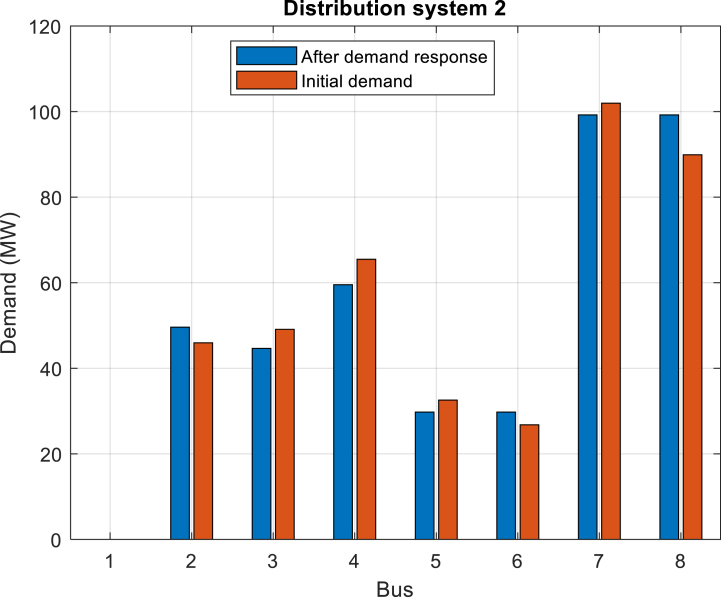
Comparison of the active load of the second distribution network in the initial state with the demand response management state in the first case.

Furthermore, Fig. 6 depicts the power purchased per hour in each of the distribution networks. Fig. 7, Fig. 8, Fig. 9 display the state of optimal charging and discharging of batteries in the first and second distribution systems and the transmission network, respectively. In Figure (9), the vertical negative axis indicates the charging of batteries in the transmission network and the vertical positive axis indicates the discharge of batteries. Fig. 10, Fig. 11 are respectively a comparison between the initial real load of the distribution network and the changed real load in the demand side management program in the first and second distribution systems. As can be seen, the orange lines in these figures illustrate the changed load in the demand-side management (DSM) program. It is known that after the implementation of the DSM, the peak load has decreased in both distribution networks. This demonstrates the positive performance of the proposed DSM.

### 4.2 Second integrated system

The second system includes the transmission network of 14 IEEE buses, in which 4 distribution networks are modeled. The first to fourth distribution networks are respectively located on buses 3, 5, 4, and 9 of the transmission network. The first distribution network, located on bus 3 of the transmission network, has 10 buses and 3 DG units on buses 2, 6, and 10. Buses 2, 5, and 10 have PV and ESS installed, and wind resources are modeled on buses 3, 6, and 9. Charging stations for electric vehicles are also planned on buses 1, 5, and 10. The second distribution network, located on bus 5 of the transmission network, consists of 5 buses and 4 lines. A distributed generation unit is located on bus 2, and PV and ESS are modeled on buses 1 and 5. The wind resource is located on bus 3, and electric vehicle charging stations are planned on buses 1 and 5. The third distribution network, located on bus 4 of the transmission network, consists of 15 buses and 14 lines. 3 DG units are located on buses 2, 7, and 10, and PV and ESS are modeled on buses 1, 5, 8, 11, and 14. Wind resources are modeled on buses 3, 8, and 11, and electric vehicle charging stations are planned on buses 1, 5, 6, 8, 10, 13, and 15. The fourth distribution network, located on bus 9 of the transmission network, consists of 33 buses and 32 lines. 3 DG units are located on buses 6, 14, and 32, and PV and ESS are modeled on buses 10, 20, and 28. Wind resources are modeled on buses 11, 19, and 26, and electric vehicle charging stations are planned on buses 5, 12, 17, 24, and 33.

In Case 1, the wind resources are located at buses 11, 19, and 26, and electric vehicle charging stations are present at buses 5, 12, 17, 24, and 33. No changes are made to the considered distribution networks in this case. The upper-level objective function has a value of $23,590, while the lower-level objective function is $7467. Additionally, the first distribution network substation purchases 17 MW, the second substation purchases 5.6 MW, the third substation purchases 23.1 MW, and the fourth substation purchases 10.6 MW of active power from the transmission network. These values provide an overview of the system's operation and coordination in Case 1. In the 14-bus transmission network, the power plant units collectively produce 1413 MW of active power over a 24-h period. To demonstrate the influence of distribution network modeling on the transmission network and validate the proposed method and model, the results of the second case are presented. In Table (3), it is evident that removing wind, solar, and fossil renewable resources from the distribution networks has had a notable impact on the transmission network. The objective function of the problem has increased from $23,590 to $26,283 in this case, indicating the significance of including renewable resources in the distribution networks.

**Table 3:** The results of the simulation of the second system in a 24-h period.

	case 1	case 2	case 3
objective function of the upper problem ($)	23590	26283	121130
objective function of the lower problem ($)	7467	11732	7467
Substation power of the first distribution network (MW)	17	25.2	17
Substation power of the second distribution network (MW)	5.6	12.9	5.6
Substation power of the third distribution network (MW)	23.1	31	23.1
Substation power of the fourth distribution network (MW)	10.6	11.8	10.6
Total transmission network power (MW)	1890	1914	1413
line outage (time)	6(14، 16–22)	4(1–2)	3(6–8، 19–21)
	12(1–5،23،24)	6(3–6، 10–16)	6(11،22،23)
	13(8–13،15)	9(22،23)	12(1–4،12،24)
		13(16–21،24)	13(5،13–18)
unit outage (time)	4(6–7)	4(9) 5(7–8)	4(9،10)

According to Table (3), in the second case, the objective function of the lower problem increased by approximately 50 % compared to the first case. This demonstrates the impact of changes in the distribution network on the objectives of the proposed two-level problem. The total power purchased by the distribution substations over a 24-h period in the second case is 25.2 MW, 12.9 MW, 31 MW, and 11.8 MW, respectively. Similarly, the total power generated by the power plant units in the 14-bus transmission network increased from 1890 MW to 1914 MW. These results highlight the influence of distribution network modifications on the overall system operation. As observed in Table 2, Table 3, the proposed model and method demonstrate favorable performance across various distribution and transmission systems. Additionally, the third case examines the influence of large-scale batteries on the transmission network. In this case, by removing the energy storage systems from the transmission network, the cost of the transmission network increases to $121,130, highlighting the significant impact of the batteries on the transmission network. These findings emphasize the importance of incorporating energy storage systems in the optimization of integrated transmission and distribution networks.

### 4.3 Third integrated system

The third system includes the transmission network with 30 IEEE buses, where 6 distribution networks are modeled. The first to sixth distribution networks are located on buses 5, 15, 20, 25, 27, and 30 of the transmission network, respectively. The first distribution network, located on the 5th bus of the transmission network, comprises 13 buses and is equipped with 2 DG units on the 7th and 9th buses. This distribution network has PV and ESS located on buses 3, 5, and 8, and wind sources modeled on buses 2, 3, and 4. Charging stations for electric vehicles are also planned on buses 10, 11, and 12. The second distribution network, located on bus 15 of the transmission network, consists of 14 buses and 13 lines. A distributed generation unit is located on bus 14, and PV and ESS are modeled on buses 10 and 11. The wind source is located on bus 5, and the charging station for electric vehicles is planned on buses 6 and 9. The third distribution network, located on bus 20 of the transmission network, consists of 22 buses and 21 lines. Three distributed generation units are located on buses 14, 16, and 18, and PV and ESS are modeled on buses 10 and 20. The wind source is located on buses 6, 14, and 22, and the charging station for electric vehicles is planned on buses 6, 9, 13, and 18. The fourth distribution network, located on the 25th bus of the transmission network, consists of 25 buses and 24 lines. Two distributed generation units are located on buses 6 and 14, and PV and ESS are modeled on buses 10, 20, and 21. The wind source is located on buses 11, 19, and 24, and the charging station for electric vehicles is planned on buses 8, 9, 10, and 16. The fifth distribution network, located on the 27th bus of the transmission network, consists of 33 buses and 32 lines. Three distributed generation units are located on buses 30, 31, and 32, and PV and ESS are modeled on buses 5, 15, and 25. The wind source is located on buses 14, 20, and 29, and the charging station for electric vehicles is planned on buses 6, 12, 15, and 18. The sixth distribution network, located on bus 30 of the transmission network, consists of 69 buses and 68 lines. Three scattered production units are located on buses 40, 50, and 60, and PV and ESS are modeled on buses 55, 56, and 59. The wind source is located on buses 34, 44, and 64, and the charging station for electric vehicles is planned on buses 6, 20, 35, and 49.

Table (4) displays the results of the third system. As observed, in both the upper and lower problems of the first and second systems, the objective function has increased by removing resources from the distribution networks. In the first case, no changes were made to the considered distribution networks, resulting in an upper level objective function value of $72,958 and a lower level objective function value of $20,139. Additionally, the amount of active power purchased by the first to sixth distribution network substations from the transmission network is 21 MW, 26 MW, 29.1 MW, 16.4 MW, 12 MW, and 14.5 MW, respectively. The total active power produced by the power plant units in the 30 bus transmission network for 24 h is 2710 MW.

**Table 4:** The results of the simulation of the third system in a 24-h period.

	case 1	case 2	case 3
objective function of the upper problem ($)	72958	81960	263326
objective function of the lower problem ($)	20139	12225	20139
Substation power of the first distribution network (MW)	21	25	21
Substation power of the second distribution network (MW)	26	32.1	26
Substation power of the third distribution network (MW)	29.1	34.7	29.1
Substation power of the fourth distribution network (MW)	16.4	19.5	16.4
Substation power of the fifth distribution network (MW)	12	14.5	12
Substation power of the sixth distribution network (MW)	14.5	17.9	14.5
Total transmission network power (MW)	2710	2735	2498
line outage (time)	16(23–24)	16(24)	16(7–9،13،17)
	22(7–10،16-21)	22(7،15،23)	22(14-10،21-23)
	35(15،22)	33(1–5،16،22)	41(1–6،18-20،24)
	41(1–6)	35(6،17–20)	
		41(10–14،21)	
unit outage (time)	3(11–14)	3(8،9)	3(10–12)

To demonstrate the impact of distribution network modeling on the transmission network and validate the proposed method and model, the results of the second case are also presented in Table (4). It is evident that removing renewable wind, solar, and fossil resources from the distribution networks has significantly affected the transmission network, increasing the objective function value from $72,958 to $81,960.

Furthermore, the third case highlights the effect of large-scale batteries on the transmission network. Removing energy storage systems from the transmission network in this case has resulted in an increased cost of $263,326, illustrating the impact of the battery on the transmission network.

### 4.4 Fourth integrated system

**Fig. 12:**
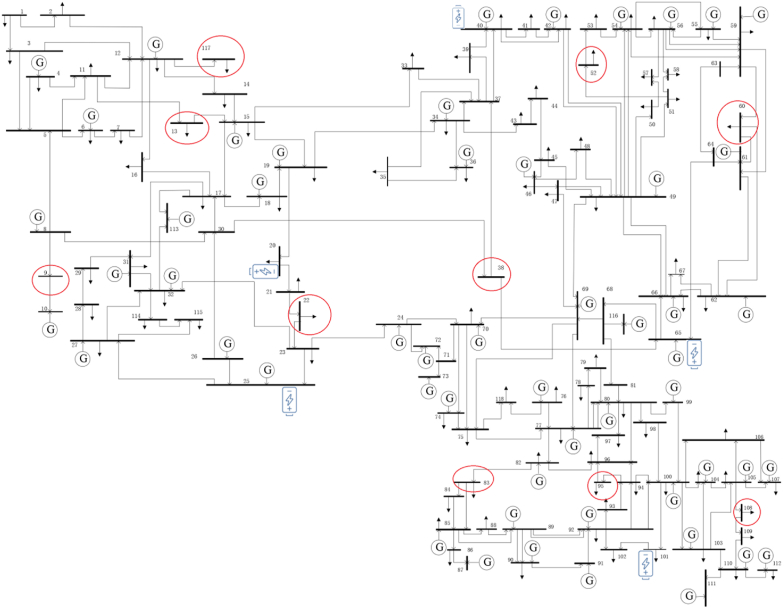
Diagram of the fourth proposed joint system.

The fourth system includes a large transmission network of 118 IEEE buses, in which 10 distribution networks are modeled. Figure (12) shows a diagram of the large network of 118 buses along with the considered distribution networks and large scale ESSs. The red circles marked in figure (11) show the modeling of distribution networks in that transmission network bus.

The distribution networks are connected to buses 9, 13, 22, 38, 52, 60, 83, 95, 108, and 117 of the transmission network, with the first to tenth distribution networks located on these buses, respectively. The first distribution network, located on bus 9 of the transmission network, comprises 13 buses and has scattered productions on buses 7 and 9. PV and ESS are situated on buses 3, 5, and 8 within this distribution network, while wind sources are modeled on buses 2, 3, and 4. Charging stations for electric vehicles are planned on buses 10, 11, and 12.

The second distribution network is positioned on bus 13 of the transmission network and consists of 14 buses and 13 lines. A distributed generation unit is located on bus 14, and PV and ESS are modeled on buses 10 and 11.

The wind source is located on bus 5, and the charging station for electric vehicles can be found on buses 6 and 9. Moving on to the third distribution network, which is situated on bus 22 of the transmission network, it consists of 22 buses and 21 lines. Within this network, there are 3 distributed production units located on buses 14, 16, and 18, while PV and ESS are modeled on buses 10 and 20. The wind source is located on buses 6, 14, and 22, and the charging station for electric vehicles can be found on buses 6, 9, 13, and 18.

As for the fourth distribution network, it is located on the 38th bus of the transmission network and comprises 25 buses and 24 lines. There are 2 distributed generation units located on buses 6 and 14, while PV and ESS are modeled on buses 10, 20, and 21. The wind source is located on buses 11, 19, and 24, and the charging station for electric vehicles can be found on buses 8, 9, 10, and 16.

The fifth distribution network, situated on the 52nd bus of the transmission network, encompasses 33 buses and 32 lines. It features three distributed production units located on buses 30, 31, and 32. Additionally, PV (Photovoltaic) and ESS (Energy Storage System) units are modeled on buses 5, 15, and 25. The wind source is situated on buses 14, 20, and 29. Moreover, charging stations for electric vehicles are present on buses 6, 12, 15, and 18. Similarly, the sixth distribution network is located on bus 60 of the transmission network. It comprises 69 buses and 68 lines, expanding the scope of the integrated system.

There are three DG units situated on buses 40, 50, and 60. Buses 55, 56, and 59 are equipped with PV and ESS models. Wind sources are placed on buses 34, 44, and 64, while the charging station for electric vehicles can be found on buses 6, 20, 35, and 49. The seventh distribution network, located on the 83rd bus of the transmission network, consists of 10 buses. Within this distribution network, there are three DGs installed on buses 2, 6, and 10. PV and ESS systems are located on buses 2, 5, and 10, while wind sources are modeled on buses 3, 6, and 9. Charging stations for electric vehicles are additionally scheduled for installation on buses 1, 5, and 10. The eighth distribution network, positioned on bus 95 of the transmission network, comprises five buses and four lines. A distributed generation unit is situated on bus 2, while PV and ESS are modeled on buses 1 and 5. The wind source is located on bus 3, and the charging station for electric vehicles is also present on buses 1 and 5. The ninth distribution network is positioned on bus 108 of the transmission network and comprises 15 buses along with 14 lines. Within this network, there are three DG units located on buses 2, 7, and 10. Additionally, PV and ESS models are implemented on buses 1, 5, 8, 11, and 14. The wind source is situated on buses 3, 8, and 11, while the electric vehicle charging station can be found on buses 1, 5, 6, 8, 10, 13, and 15. Moving on to the tenth distribution network, is situated on bus 117 of the transmission network and consists of 33 buses and 32 lines. Within this network, there are three distributed production units located on buses 6, 14, and 32. Furthermore, PV and ESS are modeled on buses 10, 20, and 28. The wind source is positioned on buses 11, 19, and 26, and the electric vehicle charging station is available on buses 5, 12, 17, 24, and 33.

Table (5) presents the results of the fourth integrated system. Similar to the first, second, and third systems, the objective function in this large system increases in both the upper and lower problems by removing resources in the distribution networks. In the first case, no changes are applied to the considered distribution networks, resulting in an upper-level objective function value of $340,980 and a lower-level objective function value of $29,586. Additionally, the active power purchased by the first to tenth distribution networks from the transmission network is 15, 31, 15.9, 11.1, 9, 13.2, 12, 4.1, 28, and 11 MW, respectively. The active power produced by the power plant units of the transmission network with 118 buses over 24 h is 4892 MW.

**Table 5:** The results of the simulation of the fourth system in a 24-h period.

	case 1	case 2	case 3
objective function of the upper problem ($)	340980	342516	752962
objective function of the lower problem ($)	29586	31981	29586
Substation power of the first distribution network (MW)	15	19.8	15
Substation power of the second distribution network (MW)	31	35.6	31
Substation power of the third distribution network (MW)	15.9	20.4	15.9
Substation power of the fourth distribution network (MW)	11.1	16.5	11.1
Substation power of the fifth distribution network (MW)	9	12.7	9
Substation power of the sixth distribution network (MW)	13.2	15.8	13.2
Substation power of the seventh distribution network (MW)	12	19.1	12
Substation power of the eighth distribution network (MW)	4.1	9.2	4.1
Substation power of the ninth distribution network (MW)	28	32	28
Substation power of the tenth distribution network (MW)	11	12.2	11
Total transmission network power (MW)	4892	4936	4320
line outage (time)	29(20–23)	96(13،21)	29(18–20)
	96(19)	133(12،22)	96(14–16)
	133(13)	152(1–5،14،20)	133(17،21)
	152(4–10،24)	160(9–11،15-19،23–24)	152(3–6،22)
	160(14–18)		160(7–9،23-24)
unit outage (time)	30(11–12) 49(1–3)	30(6–8)	30(10–13) 49(1–2)

To further demonstrate the impact of distribution network modeling on the transmission network and validate the proposed method and model, the results of the second case are also presented in Table (5). It can be observed that in the second case, the removal of renewable wind, solar, and fossil resources from the distribution networks has significantly affected the transmission network. The objective function of the problem increases from $340,980 to $342,516.

Similarly, the third case highlights the effect of large-scale batteries on the transmission network. After removing the energy storage systems from the transmission network, the cost of the transmission network increases to $752,962, indicating the impact of the batteries on the transmission network.

### 4.5 Comparison

In this section, we compare the proposed method with an evolutionary method, as described in Ref. [40]. Table (6) presents a comparison between the solution time and the cost function of different systems implemented using the proposed method and the evolutionary method. It can be observed that the proposed method outperforms the evolutionary method in terms of speed, as it is faster in solving the problem. Additionally, the solutions obtained using the proposed method show an improvement of nearly 2.5 % compared to the evolutionary method in terms of the cost function. Further analysis of Table (6) reveals that the proposed method is significantly superior to the evolutionary methods both in terms of solution time and the quality of the obtained solutions.

**Table 6:** Comparison of the proposed method with the evolutionary method.

	Proposed	Evolutionary method
Time to solve the first system (seconds)	2078	2260
Time to solve the second system (seconds)	17121	17950
Time to solve the third system (seconds)	29590	30870
Time to solve the fourth system (seconds)	59344	60878
Cost function of the first system (dollars)	6951	7604
Cost function of the second system (dollars)	23590	27745
Cost function of the third system (dollars)	72958	78924
Cost function of the fourth system (dollars)	340980	343440

### 4.6 Uncertainty sensitivity analysis

In this dedicated section, we systematically examine the impact of varying the number of uncertainty scenarios associated with renewable energy resources and electric vehicles on both the objective function and the computational efficiency of the problem-solving process. Through a methodical exploration of different scenario quantities, we aim to provide valuable insights into the sensitivity of our model to the representation of uncertainties, offering a nuanced understanding of its performance under diverse conditions. The objective of this analysis is twofold. First, we investigate how the number of uncertainty scenarios influences the objective function, elucidating the relationship between the model's outcomes and the granularity of uncertainty representation. By systematically adjusting the scenario count, we can discern patterns in the objective function values, thereby shedding light on the trade-off between accuracy and computational complexity. Simultaneously, we assess the duration of the problem-solving process in relation to the number of uncertainty scenarios. This analysis not only contributes to our understanding of the computational efficiency of the proposed model but also provides practical insights for decision-makers who may need to balance the accuracy of results with the computational resources required. Through this detailed exploration, we endeavor to contribute not only to the understanding of our specific model's behavior but also to the broader discourse on the balance between accuracy and computational efficiency in addressing uncertainties within the realm of renewable energy resources and electric vehicles.

According to table (7) with the increase in the number of scenarios, the solution time of each system also increases. This is because the optimization algorithm has to consider more possible combinations of parameter values. The time to solve the first system increases from 2078 s with 20 scenarios to 2459 s with 200 scenarios. This is an increase of approximately 18 %. The time to solve the fourth system increases from 59344 s with 20 scenarios to 70951 s with 200 scenarios. This is an increase of approximately 20 %. The cost function of each system also increases as the number of scenarios increases. This is because the optimization algorithm is able to find better solutions with more scenarios. The cost function of the first system increases from $6951 with 20 scenarios to $6957 with 200 scenarios. This is an increase of approximately 0.1 %. The cost function of the fourth system increases from $340980 with 20 scenarios to $340988 with 200 scenarios. This is an increase of approximately 0.02 %. In general, the benefits of using more scenarios outweigh the costs. However, there is a point of diminishing returns. With too many scenarios, the optimization algorithm becomes too slow and the cost function increases only marginally. The optimal number of scenarios will depend on the specific application.

**Table 7:** Effect of the number of scenarios on objective functions and problem-solving time.

Number of scenarios	20	50	100	200
**Time to solve the first system (sec)**	2078	2197	2297	2459
**Time to solve the second system (sec)**	17121	17924	18829	20141
**Time to solve the third system (sec)**	29590	31047	32641	35102
**Time to solve the fourth system (sec)**	59344	62330	65210	70951
**Cost function of the first system ($)**	6951	6961	6962	6957
**Cost function of the second system ($)**	23590	23597	23599	23595
**Cost function of the third system ($)**	72958	72963	72966	72961
**Cost function of the fourth system ($)**	340980	340989	340991	340988

**Fig. 13:**
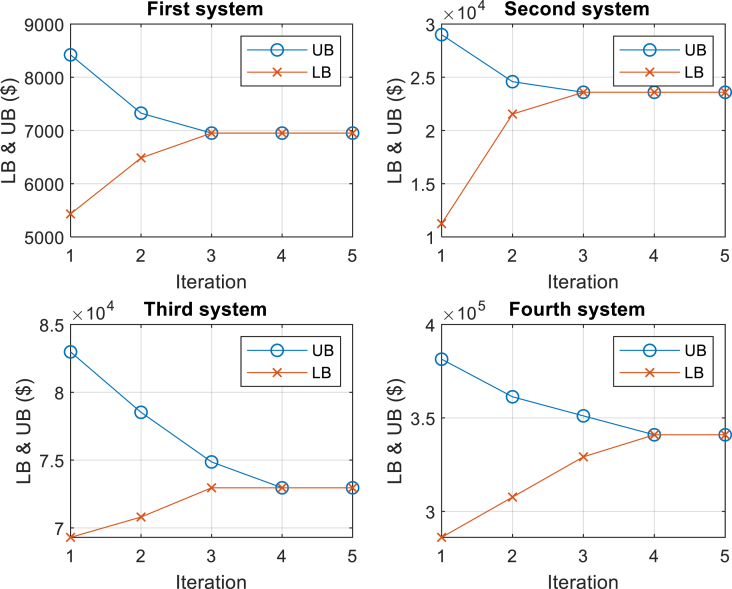
The convergence of the proposed algorithm.

Fig. 13 demonstrates the convergence of the proposed algorithm in each considered test system. It can be seen that in most systems, the proposed algorithm has reached convergence in 3 iterations, which illustrates the superiority of the proposed algorithm.

## 5 Conclusion

In conclusion, this research provides a significant contribution to the optimization of electric grids by adopting a holistic approach to enhance flexibility and achieve smart grid objectives. The comprehensive investigation into the impact of optimal Energy Storage System (ESS) operation on both transmission and distribution networks sets this study apart from previous research. The proposed bi-level stochastic model for integrated energy management considers renewable energy, demand-side management (DSM), transmission network, and distribution network as interconnected components, offering a more realistic representation of the complex interactions within the grid. The utilization of an exact method based on reformulation and decomposition techniques to address binary variables ensures the derivation of globally optimal solutions. The evaluation of the proposed model through various integrated systems demonstrates its efficiency. Notably, the incorporation of ESS in the distribution grid leads to a substantial 13 % decrease in distribution network costs. Moreover, the presence of large batteries in the transmission grid yields an impressive 83 % reduction in transmission network costs, highlighting the significant cost reduction benefits associated with the integration of energy storage systems. These findings underscore the importance of considering both transmission and distribution networks in the optimization of ESS operation, emphasizing the potential for substantial cost savings and improved efficiency. As the global energy landscape continues to evolve, with an increasing focus on renewable resources and grid modernization, the insights provided by this research are invaluable for policymakers, utility operators, and researchers working towards the development of sustainable and resilient energy infrastructure.

To further advance the scope of this paper, the following recommendations are suggested.

1) Incorporate the modeling of natural gas distribution and transmission networks to enhance the accuracy of modeling gas-fired power plants, particularly in light of the global challenges related to the depletion of fossil fuels. This addition would contribute to a more comprehensive understanding of the interplay between energy sources and power generation.
2) Integrate the problem of load forecasting using machine learning techniques to improve the precision of load modeling for future timeframes. By leveraging advanced prediction methodologies, the paper can offer a more robust analysis of the dynamic nature of energy demand, providing valuable insights for planning and optimizing power systems in the face of evolving consumption patterns.

## CRediT authorship contribution statement

**Meysam Khani:** Data curation, Conceptualization. **Mahmoud Samiei Moghaddam:** Writing – review & editing, Writing – original draft, Visualization, Validation, Supervision, Project administration. **Tohid Noori:** Software, Resources. **Reza Ebrahimi:** Investigation, Funding acquisition, Formal analysis.

## Ethics approval and consent to participate

“Not applicable”

## Consent for publication

“Not applicable”Availability of data and materials

Data is available on request. To request data, contact the corresponding author Mahmoud Samiei Moghaddam by e-mail: samiei352@yahoo.com at Islamic Azad University, Damghan branch, Damghan, Iran.

## Funding

The authors declare that they have received no funding.

## Declaration of competing interest

The authors declare that they have no known competing financial interests or personal relationships that could have appeared to influence the work reported in this paper.
